# Polymer Nanoparticles in Medical Applications—Future Directions

**DOI:** 10.3390/nano16100630

**Published:** 2026-05-19

**Authors:** Barbara Zawidlak-Węgrzyńska, Joanna Rydz

**Affiliations:** 1 Department of Chemistry, Faculty of Medicine in Zabrze, Academy of Silesia, 40-555 Katowice, Poland; 2 Centre of Polymer and Carbon Materials, Polish Academy of Sciences, 41-819 Zabrze, Poland; jrydz@cmpw-pan.pl

**Keywords:** polymer-based nanomaterials, nanoparticles, diagnostics, theranostics, nanosponges, regenerative medicine, personalized medicine, artificial intelligence, biomedicine

## Abstract

Polymer-based nanoparticle systems have emerged as a versatile platform for advancing precision medicine by enabling controlled, targeted, and multifunctional drug delivery. This narrative review synthesizes recent progress in the design, functionalization, and clinical translation of polymer-based nanoparticles, with a focused scope on drug delivery, diagnostics, theranostics, nanosponges, and regenerative medicine. Specifically, it highlights three key insights: (i) surface engineering strategies, including ligand conjugation and stealth coatings, substantially enhance targeting specificity and reduce off-target toxicity; (ii) stimulus-responsive polymers enable spatiotemporally controlled drug release, improving therapeutic outcomes in complex disease microenvironments; and (iii) integration with artificial intelligence (AI) supports the rational design of personalized nanomedicines based on patient-specific molecular profiles. The innovative nature of this review lies in its comprehensive approach, which combines material design parameters with clinical outcomes and the barriers to implementation. Despite significant progress, serious challenges remain, including scalable and reproducible manufacturing, regulatory harmonization, and comprehensive long-term biosafety assessment. In the future, the priority should be to develop reliable manufacturing processes, a harmonized regulatory framework, and data-driven, clinically validated design methodologies. Overall, polymer-based nanoparticles are poised to redefine targeted therapy, but their clinical impact will depend on bridging the gap between laboratory innovation and scalable, safe, and personalized medical applications.

## 1. Introduction

Over the past few decades, polymer nanoparticles have emerged as one of the most promising platforms in modern biomedical science, bridging the disciplines of materials engineering, chemistry, pharmacology, and clinical medicine [1]. These nanoscale structures, typically ranging from 10 to 1000 nm in diameter, are composed of natural or synthetic polymers designed to encapsulate, adsorb, or conjugate therapeutic agents [2]. Their small size, tunable surface properties, and capacity for controlled drug release have positioned them at the forefront of advanced drug delivery systems. As healthcare shifts toward precision medicine, minimally invasive therapies, and biologically targeted interventions, polymer nanoparticles are becoming increasingly central to the future of medical innovation [3].

Traditional drug delivery methods often suffer from significant limitations, including poor solubility of therapeutic agents, rapid systemic clearance, off-target toxicity, and limited bioavailability. Many potent drugs fail in clinical translation not because of inefficacy but because of challenges in safe and effective delivery. Polymer nanoparticles address these issues by improving pharmacokinetics, protecting fragile molecules from degradation, enhancing tissue penetration, and enabling controlled or stimulus-responsive release [4]. By modifying the chemical structure and surface functionality of polymers, researchers can tailor nanoparticles to specific biological environments, optimizing therapeutic outcomes while minimizing adverse effects [5,6,7].

In oncology, polymer nanoparticles are revolutionizing targeted therapy by enabling more precise delivery of anticancer agents while minimizing systemic toxicity [8,9]. Conventional chemotherapy distributes cytotoxic drugs throughout the body, damaging healthy tissues and causing severe side effects. A major mechanism underlying passive targeting is the enhanced permeability and retention (EPR) effect, where the abnormal architecture of tumor vasculature—characterized by wide fenestrations and poor lymphatic drainage—facilitates the preferential accumulation of polymer nanoparticles within tumor tissues. However, reliance on the EPR effect alone can be insufficient due to tumor heterogeneity and variability among patients [10,11]. To address this limitation, active targeting strategies have been developed. These involve functionalizing the surface of polymer nanoparticles with ligands such as monoclonal antibodies, peptides, aptamers, or small molecules that specifically bind to overexpressed receptors on cancer cells (e.g., folate receptors, HER2 (human epidermal growth factor receptor 2), and transferrin receptors). This receptor-mediated recognition enhances cellular uptake and improves intracellular drug delivery efficiency [12,13]. These innovations open new possibilities for combination therapies, co-delivery of multiple drugs, and personalized cancer treatment strategies.

Beyond targeting mechanisms, the performance of polymer nanoparticles is strongly influenced by the compatibility between the polymer matrix and the nanoparticulate components, particularly in composite or hybrid systems. Compatibility governs nanoparticle stability, drug encapsulation efficiency, and release behavior. It is largely dictated by physicochemical factors such as polymer polarity, crystallinity, molecular weight, and intermolecular interactions (e.g., hydrogen bonding, van der Waals forces, and electrostatic interactions). Hydrophilic–hydrophobic balance is especially critical: amphiphilic block co-polymers, for example, can self-assemble into core–shell structures, where hydrophobic domains encapsulate drugs, while hydrophilic shells, often poly(ethylene glycol) (PEG), stabilize the system in biological fluids and reduce opsonization [14,15].

During synthesis, polymer nanoparticles can be produced using techniques such as nanoprecipitation, emulsion–solvent evaporation, salting-out, or microfluidic-assisted assembly. Each method influences particle size distribution, morphology, and internal structure. Kinetic factors—such as solvent diffusion rate, polymer concentration, and mixing intensity—have a decisive role in nucleation and growth processes. Rapid solvent displacement, for instance, can lead to smaller and more uniform polymer nanoparticles, whereas slower kinetics may promote phase separation or heterogeneous structures [16]. Additionally, the presence of surfactants or stabilizers is essential to controlling interfacial energy and preventing uncontrolled aggregation during formation.

Aggregation and dispersion behavior are central challenges in the design of polymer nanoparticles, both during synthesis and in their final application environments. Nanoparticles have a natural tendency to aggregate due to high surface energy, which can compromise colloidal stability, alter biodistribution, and reduce targeting efficiency. To mitigate this, steric stabilization (e.g., PEGylation) and electrostatic stabilization (via charged surface groups) are commonly employed. Proper dispersion within polymer matrices or coatings is particularly important for applications such as implantable drug delivery systems or surface-functionalized medical devices. Uniform dispersion ensures consistent drug release profiles and mechanical integrity, whereas aggregation can lead to local defects, burst release, or reduced bioavailability [17].

In composite systems, interfacial interactions between polymer nanoparticles and surrounding matrices determine overall performance. Strong interfacial adhesion can enhance mechanical stability and prevent nanoparticle leaching, while weak interactions may facilitate faster drug diffusion but reduce structural integrity. Advanced strategies, such as surface functionalization or the grafting of polymer chains onto nanoparticles, are used to improve compatibility and dispersion within host materials [18].

Beyond oncology, polymer nanoparticles are advancing therapeutic approaches in infectious diseases, cardiovascular disorders, neurological conditions, and regenerative medicine. In vaccine development, nanoparticle carriers enhance antigen stability and promote stronger immune responses by improving antigen presentation and controlled release. In neurological applications, polymer nanoparticles show potential for crossing the blood–brain barrier, a longstanding obstacle in treating central nervous system diseases such as Alzheimer’s, Parkinson’s, and brain tumors [19]. Their ability to transport small molecules, proteins, RNA, and gene-editing tools into protected biological compartments marks a transformative shift in therapeutic design. The rapid progress in nanotechnology has also been closely linked with breakthroughs in molecular biology and genetic medicine. The success of lipid-based nanoparticles in mRNA vaccine platforms has highlighted the broader potential of polymeric nanoparticles in nucleic acid delivery. Inspired by these developments, polymer-based nanoparticles are being engineered for safer and more stable gene therapy applications [20]. Compared with viral vectors, polymer systems offer reduced immunogenicity, flexible design, and scalable manufacturing. In parallel, gene-editing technologies such as CRISPR (clustered regularly interspaced short palindromic repeats) have further intensified interest in nanoparticle-based delivery strategies. Polymer nanoparticles are being investigated as potential vehicles for the transport of CRISPR-associated components, with the aim of improving delivery precision and reducing off-target effects. However, most studies remain at the preclinical stage, and further work is required to demonstrate safety, efficiency, and clinical translatability [21]. Looking ahead, the future directions of polymer nanoparticles in medicine are shaped by several emerging trends. One of the most important areas is the development of stimulus-responsive or smart nanoparticles that respond to changes in pH, temperature, enzymatic activity, or redox conditions. These systems enable site-specific drug release, ensuring that therapeutic agents are activated only within diseased tissues [22]. For instance, tumor microenvironments often exhibit acidic pH and elevated enzyme levels, which can be exploited for selective drug activation. Such intelligent systems enhance efficacy while reducing systemic toxicity. Another promising direction involves multifunctional or theranostic nanoparticles that combine therapeutic and diagnostic capabilities within a single platform [23]. By incorporating imaging agents such as fluorescent dyes, magnetic particles, or radiolabels, polymer nanoparticles can enable the real-time monitoring of drug distribution and treatment response. This integration of therapy and diagnostics supports the growing paradigm of personalized medicine, where treatments are adapted based on individual patient profiles and dynamic biological feedback.

Advances in polymer chemistry and nano-fabrication techniques are also expanding the design possibilities of nanoparticles. Techniques such as microfluidics, self-assembly, and three-dimensional (3D) printing allow for precise control over size, shape, and surface architecture [24]. Research indicates that nanoparticle geometry significantly influences cellular uptake, biodistribution, and immune interaction. Tailoring these parameters may optimize clinical performance and overcome biological barriers that currently limit therapeutic efficiency. Despite remarkable progress, several challenges remain before polymer nanoparticles can achieve their full clinical potential. Issues related to large-scale manufacturing, long-term safety, regulatory approval, and reproducibility must be carefully addressed. Biological complexity, including immune system interactions and interpatient variability, adds further hurdles [25]. Future research must focus not only on innovative design but also on translational feasibility, cost-effectiveness, and ethical considerations. Future directions for polymer nanoparticles in medicine include enhanced theranostics, personalized medicine, and smart drug delivery systems. Important areas of focus are developing more precise and responsive nanoparticles, improving manufacturing scalability and reproducibility, and expanding their use in areas like tissue engineering and advanced diagnostics. The literature search strategy and study selection criteria are shown in Table 1.

## 2. The Method of Administration of Nanoparticles and Formulation Implications

### 2.1. Overview of Administration Pathways

The route of administration is a key determinant of nanoparticle (NP) performance, governing biodistribution, pharmacokinetics, cellular uptake, and therapeutic efficacy. Nanoparticles can be delivered via parenteral, oral, pulmonary, nasal, dermal, and ocular routes, each presenting unique biological barriers that directly influence formulation design. Consequently, NP physicochemical properties—such as size, surface charge, hydrophobicity, and stability—must be tailored according to the intended administration pathway to ensure optimal performance and safety [5,6,7].

### 2.2. Parenteral Administration (Intravenous, Intramuscular, Subcutaneous, and Intradermal)

Parenteral administration remains the most clinically established route for nanoparticle-based therapeutics, particularly for cancer therapy, vaccines, and gene delivery systems. Intravenous (IV) injection enables immediate systemic distribution but requires strict control of particle size (typically < 200 nm), sterility, and colloidal stability to prevent aggregation and unintended embolism. Surface modifications such as PEGylation or biomimetic coatings are widely used to reduce opsonization and prolong circulation time [6,8].

Subcutaneous (SC) and intramuscular (IM) delivery enable depot formation, allowing for sustained release of encapsulated drugs. These routes are particularly useful for long-acting formulations such as peptide therapeutics and vaccines. Intradermal (ID) delivery has gained increasing importance due to the high density of antigen-presenting cells in the dermis, making it highly effective for immunotherapy and vaccine delivery. However, these routes require careful control of particle degradation kinetics and local tissue compatibility [5,9].

### 2.3. Oral Administration

Oral delivery is the most patient-friendly route but remains challenging for nanoparticle systems due to enzymatic degradation, acidic gastric conditions, and epithelial barriers in the gastrointestinal tract. To overcome these limitations, nanoparticles are often engineered with protective coatings such as polysaccharides, lipids, or pH-sensitive polymers that enable controlled release in the intestine.

Mucoadhesive and mucus-penetrating nanoparticles have been developed to enhance intestinal residence time and improve absorption. Nevertheless, oral bioavailability of nanoparticle-based macromolecular drugs remains limited, and efficient systemic delivery requires optimization of particle size (<200 nm), surface neutrality, and enzymatic resistance [7,19].

### 2.4. Pulmonary and Nasal Administration

Pulmonary delivery offers a large absorptive surface area and thin epithelial barrier, enabling both local and systemic drug delivery. Nanoparticles intended for inhalation are often formulated as nano-aggregates or embedded in microparticles to achieve an aerodynamic diameter of 1–5 µm for optimal lung deposition. Once deposited, nanoparticles can dissolve or be internalized by alveolar macrophages, influencing drug release kinetics [6,20].

Nasal administration provides a non-invasive route with potential for direct brain targeting via the olfactory and trigeminal pathways. However, mucociliary clearance limits residence time, requiring mucoadhesive or mucus-penetrating formulations. These strategies are increasingly explored for neurodegenerative disease therapies and vaccine delivery [9,20].

### 2.5. Dermal and Transdermal Administration

The skin represents a highly protective barrier due to the stratum corneum, limiting passive nanoparticle penetration. Intradermal injection bypasses this barrier and enables direct access to immune-rich dermal layers, making it highly suitable for vaccine delivery.

Transdermal nanoparticle systems rely on chemical enhancers, lipid-based carriers, or microneedle-assisted delivery to facilitate penetration. Particle size, surface charge, and deformability play critical roles in determining penetration efficiency through intercellular or appendageal pathways [8,26].

### 2.6. Ocular Administration

Ocular drug delivery is limited by tear turnover, corneal epithelium tight junctions, and limited drug retention time. Nanoparticles improve ocular bioavailability by enhancing corneal penetration and prolonging precorneal residence time. Lipid-based and polymeric nanoparticles are widely used to deliver both anterior and posterior segment therapies.

Mucoadhesive and in situ gelling systems further enhance retention and controlled release, making nanoparticle systems particularly promising for chronic ocular diseases such as glaucoma and macular degeneration [7,19].

### 2.7. Formulation Implications of Administration Routes

Each route of administration imposes distinct constraints on nanoparticle design. Systemic routes (IV, IM, and SC) require high colloidal stability, immune evasion strategies, and controlled pharmacokinetics. Oral and nasal routes demand resistance to enzymatic degradation and mucus penetration, while pulmonary delivery requires aerodynamic optimization for deep lung deposition.

Across all routes, surface engineering (e.g., PEGylation and ligand conjugation), size optimization, and release modulation are critical formulation strategies. These design principles ensure that nanoparticles can overcome biological barriers while maintaining therapeutic efficacy and minimizing toxicity [5,6,7,26].

## 3. Advancements in Diagnostics, Theranostics and Personalized Medicine

### 3.1. Advanced Diagnostics—Polymer Nanoparticles in Imaging Techniques

Conventional imaging contrast agents often suffer from rapid clearance, limited targeting ability, and potential toxicity. Polymer nanoparticles overcome these limitations by enhancing bioavailability, protecting imaging payloads, and enabling surface functionalization for active targeting [26]. Consequently, polymer nanoparticles (PNPs) have emerged as versatile platforms in biomedical imaging due to their biocompatibility with many organisms, tunable physicochemical properties, and multifunctional capabilities. They enhance diagnostic modalities such as magnetic resonance imaging (MRI), computed tomography (CT), positron emission tomography (PET), single-photon emission computed tomography (SPECT), optical imaging, and ultrasound by improving contrast, targeting efficiency, and circulation time [27,28]. Their ability to combine multiple imaging agents within a single nanoplatform enables multimodal imaging applications. Fluorescence imaging represents the most advanced and widely explored application of polymeric nanoparticles. In particular, conjugated polymer nanoparticles and polymer dots exhibit high molar absorption, exceptional photostability and precisely tunable emission spectra, including emission in the near-infrared (NIR) and second near-infrared (NIR-II) regions. These properties enable high-resolution, real-time imaging at the cellular and small-animal levels. Furthermore, their surfaces can be readily modified with targeting ligands, facilitating the selective imaging of pathological tissues. However, the limited penetration depth of light in biological tissues (typically < 1 cm) and interference from tissue autofluorescence significantly restrict their applicability in deep-tissue clinical imaging. Consequently, most fluorescence-based polymeric nanoparticle systems remain in preclinical development or are being explored in early-stage translational applications such as fluorescence-guided surgery [29,30,31,32,33].

MRI-based polymeric nanoparticle systems offer complementary advantages, including deep-tissue penetration, high spatial resolution, and the absence of ionizing radiation. In these systems, polymeric nanoparticles often function as carriers for contrast-generating agents such as superparamagnetic iron oxide nanoparticles (SPIONs) or gadolinium complexes. The polymer matrix enhances biocompatibility with many organisms, circulation time, and targeting capability. Nevertheless, MRI suffers from relatively low sensitivity compared with optical and nuclear imaging modalities, necessitating high local concentrations of contrast agents. Additionally, concerns regarding long-term toxicity (particularly for gadolinium-based systems) and the complexity of synthesizing stable polymer–inorganic hybrids hinder clinical translation. Consequently, most polymeric nanoparticles showing activity in MRI studies remain at the preclinical stage, and progress in their clinical evaluation and implementation is limited. [5,8].

Nuclear imaging modalities, such as PET and SPECT, provide exceptional sensitivity while enabling quantitative, whole-body imaging with high diagnostic accuracy. Radiolabeled polymeric nanoparticles enable the precise tracking of biodistribution, accumulation, and clearance profiles in vivo, making them particularly valuable for pharmacokinetic studies. However, the incorporation of radionuclides introduces challenges related to radiochemical stability, short isotope half-lives, and radiation safety. These factors complicate large-scale production and regulatory approval. Although several studies have demonstrated the feasibility of polymeric nanoparticle-based PET/SPECT imaging in preclinical models, clinical translation has been limited to a small number of investigational systems [9,19].

Multimodal imaging platforms based on polymeric nanoparticles have gained increasing attention, as they integrate the complementary strengths of different imaging techniques. For example, hybrid systems combining fluorescence imaging with MRI or PET enable simultaneous high sensitivity and high spatial resolution while also supporting theranostic applications such as image-guided drug delivery. These systems typically involve sophisticated nanoparticle architectures incorporating multiple functional components, including optical dyes, magnetic cores, and radiolabels. While multimodal polymeric nanoparticles demonstrate significant promise in preclinical studies, their clinical translation is impeded by synthetic complexity, reproducibility issues, and challenges in meeting regulatory requirements for multi-component nanomedicines [6,8].

For example, advanced imaging modalities, including dynamic contrast-enhanced MRI and PET imaging with nanoparticle tracers, enable in vivo assessment of nanoparticle distribution [34]. Ilosvai et al. developed polyvinylpyrrolidone (PVP)-coated zinc ferrite (ZnFe_2_O_4_ NH_2_) nanoparticles as MRI contrast agents [35]. The magnetic nanoparticles were stabilized in a polymer matrix and tested in mice, showing effective contrast enhancement in the liver at low doses without observed toxicity. The study emphasizes colloidal stability and potential pharmaceutical applicability of polymer-encapsulated magnetic nanoparticles. Xu et al. reported bimodal MRI/fluorescence nanoparticles designed to target gastrin-releasing peptide receptors in aggressive prostate cancer. These ultra-small polymer-coated iron oxide nanoparticles offered strong T_2_ (transverse relaxation time) MRI contrast and high receptor specificity in both in vitro and in vivo models, underscoring polymer surface modification to enhance MRI contrast and targeting [36]. In another study, poly(butyl cyanoacrylate)-coated magnetic nanocomposites, magneto-polymeric nanocomposite carriers composed of PEGylated nanoflowers cores and poly(butyl cyanoacrylate shells, were investigated, showing that polymer shell thickness significantly affects both T_2_ MRI contrast and magnetic hyperthermia performance. This highlights polymer engineering as a tool to improve multifunctional nanoparticles [37]. Table 2 summarizes the use of polymer nanoparticles in imaging techniques.

### 3.2. Integrated Theranostics

Integrated theranostics—where a single nanosystem combines therapeutic and diagnostic functions—has become a central strategy in precision medicine. Polymeric nanoparticles are particularly well suited for this purpose due to their structural versatility, biocompatibility with many organisms, and ability to incorporate multiple functionalities within a single platform. These nanoparticles are often constructed from amphiphilic block co-polymers that self-assemble into core–shell structures, enabling the encapsulation of hydrophobic drugs in the core while hydrophilic shells enhance circulation stability and reduce immune recognition [45].

A key feature of polymer-based theranostic nanoparticles is their ability to co-deliver therapeutic agents alongside imaging probes [46,47]. Common therapeutic payloads include chemotherapeutic drugs such as doxorubicin or paclitaxel, while diagnostic components may involve fluorescent dyes, magnetic resonance imaging contrast agents (e.g., gadolinium complexes), or radionuclides. This dual functionality allows clinicians to visualize nanoparticle distribution, accumulation, and drug release in real time, which provides critical insights into treatment effectiveness [48].

Stimulus-responsive design further enhances the precision of these systems. Polymeric nanoparticles can be engineered to respond to tumor-specific conditions such as acidic pH, elevated glutathione levels, or enzyme activity. These triggers induce structural changes or bond cleavage, leading to controlled drug release specifically at the disease site. Importantly, many systems are designed as “self-reporting” polymeric nanoparticles [49,50], where the activation of the imaging signal is directly coupled with drug release. For example, fluorescence resonance energy transfer (FRET)-based systems [51] remain quenched during circulation but emit fluorescence upon nanoparticle disassembly, enabling the real-time monitoring of therapeutic activation. Light-based theranostics, including techniques such as photodynamic therapy (PDT) and photothermal therapy (PTT), use photosensitive agents to target and eliminate cancer cells. In the PDT technique, for example [52], a photosensitizing drug produces reactive oxygen species (ROS) when activated by a specific wavelength of light, inducing cancer cell death. This technique is effective in treating skin, esophageal, and lung cancer.

Targeting ligands are frequently incorporated to improve specificity. Molecules such as antibodies, peptides, or small ligands (e.g., folic acid or glycyrrhizic acid) [53] bind selectively to overexpressed receptors on cancer cells, enhancing nanoparticle uptake and minimizing off-target toxicity [54,55]. This targeted approach ensures that both therapy and diagnostic signals are localized, improving treatment efficacy and the accuracy of imaging readouts.

However, across the literature, the majority of reported systems (~60–75%) are confined to in vitro proof-of-concept studies, demonstrating cellular uptake, imaging capability, or preliminary therapeutic effects without in vivo validation. A smaller fraction (~20–30%) advances to small-animal in vivo imaging, typically focusing on tumor accumulation or enhanced contrast, but without rigorous therapeutic endpoints. Only ~5–15% of systems achieve meaningful in vivo theranostic validation, defined as combined imaging and therapeutic efficacy in murine models with demonstrable tumor suppression. Fewer than 5% progress toward preclinical translational benchmarks such as pharmacokinetics, biodistribution clearance, repeated dosing, and systemic toxicity evaluation, and clinical translation remains exceedingly rare (<1%) [56,57,58].

This distribution reflects a persistent “proof-of-concept inflation” within nanotheranostics, where publication output is dominated by early-stage demonstrations rather than clinically relevant validation. Key barriers include poor degradability of polymer backbones, accumulation in the reticuloendothelial system, limited reproducibility, and lack of standardized translational criteria. Consequently, while targeted polymeric nanoparticles offer strong modularity for imaging and therapy integration, their progression beyond initial in vivo studies remains constrained. Addressing these limitations is essential to bridging the gap between preclinical nanotechnology design and clinically translatable theranostic platforms.

In recent years, artificial intelligence (AI) has emerged as a powerful tool in advancing integrated theranostics [59,60]. AI algorithms, including machine learning and deep learning models, are increasingly used to optimize nanoparticle design by predicting physicochemical properties, drug loading efficiency, and biological interactions. These models can analyze large datasets to identify optimal polymer compositions and architectures, significantly reducing experimental trial and error.

AI also plays a critical role in interpreting diagnostic data generated by theranostic nanoparticles [61]. Advanced image analysis techniques enable automated quantification of nanoparticle accumulation, drug release, and tumor response from imaging modalities such as MRI, CT, and fluorescence imaging. This facilitates real-time, data-driven decision making in clinical settings. Moreover, AI can integrate multimodal data—including imaging, genomic, and clinical information—to support personalized treatment strategies [62]. Table 3 summarizes the role of AI in theranostics.

**Figure 1:**
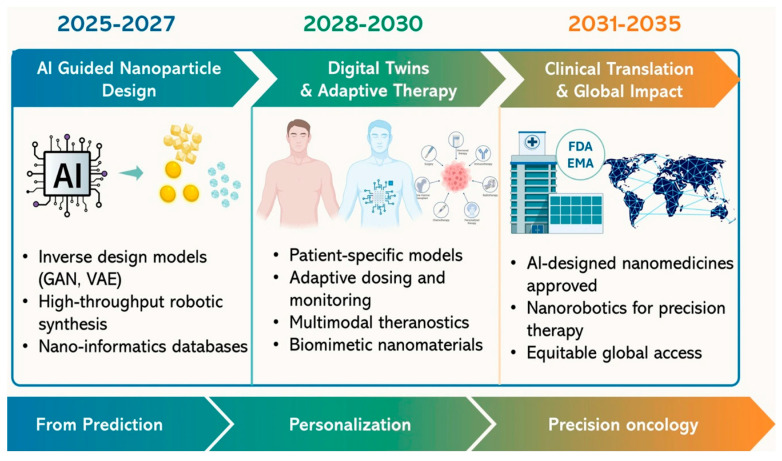
The projected development of nanotheranostics integrated with AI. Originally published in ref. [62] under CC BY 4.0 license. Copyright [2026] Molecular Cancer.

Despite significant progress, several challenges remain. The complexity of multifunctional nanoparticle systems can hinder large-scale manufacturing and regulatory approval. Potential toxicity of certain imaging agents and long-term safety concerns must also be addressed. Additionally, integrating AI into clinical workflows requires robust validation, standardization, and data security measures. Figure 1 presents a projected 2025–2035 roadmap outlining the evolution of AI-integrated nanotheranostics. The first phase (2025–2027) focuses on AI-guided nanoparticle design, where generative models and automated synthesis platforms accelerate material discovery and contribute to the development of standardized nano-informatics databases. The second phase (2028–2030) shifts toward digital twins and adaptive therapy, integrating patient-specific computational models with real-time monitoring, multimodal imaging, and biomimetic nanoplatforms to enable more personalized treatment strategies. The final phase (2031–2035) anticipates clinical translation and broader implementation, with potential regulatory approval of AI-assisted nanomedicines, advances in nanorobotics for targeted drug delivery, and efforts to improve global accessibility.

### 3.3. Personalized Medicine

**Figure 2:**
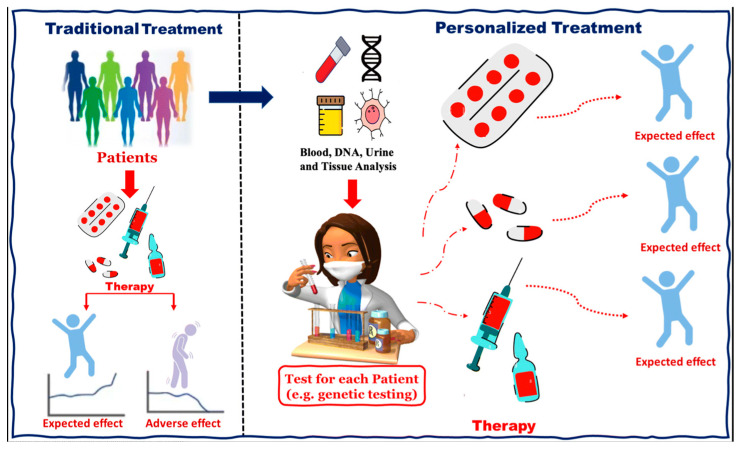
Differences between traditional medicine and personalized medicine. Originally published in ref. [71] under CC BY 3.0 license. Copyright [2025] RSC Pharmaceutics.

Personalized medicine aims to move beyond the “one-size-fits-all” paradigm by tailoring therapeutic strategies to the biological characteristics of individual patients, an approach enabled by advances in genomics, biomarker discovery, and data-driven diagnostics (Figure 2) [71]. The convergence of nanotechnology, high-resolution molecular profiling, and AI has opened the possibility of designing patient-specific nanoparticle drug delivery systems. However, while the conceptual framework is compelling, the degree of clinical implementation varies widely across applications [72,73,74,75].

Polymeric nanoparticles are particularly well suited for personalization due to their highly tunable physicochemical properties, including size, surface charge, morphology, and functionalization, which directly influence biodistribution, cellular uptake, and drug release kinetics [76,77]. In practice, patient stratification already informs nanoparticle design in several contexts. For example, in HER2-positive breast cancer, nanoparticles functionalized with anti-HER2 ligands (such as trastuzumab-conjugated systems) have demonstrated enhanced uptake in tumors overexpressing the receptor, with patient selection guided by immunohistochemical profiling [78]. Similarly, folate receptor-targeted nanoparticles have been explored in ovarian and lung cancers, where receptor expression varies across patient subgroups [79]. These examples illustrate how biomarker-defined populations can guide the selection of targeting ligands and improve therapeutic specificity.

Beyond receptor targeting, features of the tumor microenvironment provide additional axes for personalization. For instance, pH-responsive polymeric nanoparticles have been engineered to exploit the mildly acidic extracellular environment of many solid tumors, enabling preferential drug release at pH ~6.5 while remaining stable in circulation [80]. Since tumor acidity varies between patients and cancer types, imaging-based or biopsy-derived pH measurements can inform the selection of such systems. Enzyme-responsive nanoparticles offer another example: carriers incorporating matrix metalloproteinase (MMP)-cleavable linkers have shown improved penetration and drug release in tumors with high MMP activity, such as glioblastoma [81]. In these cases, the molecular profiling of tumor tissue directly informs nanoparticle composition.

Advanced nanoparticle characterization remains a critical enabler of this personalization. Techniques such as dynamic light scattering (DLS), electron microscopy, nanoparticle tracking analysis, and zeta potential measurements provide detailed information on particle size distribution, stability, and surface properties [77,82]. When combined with high-throughput screening platforms, these methods allow for systematic evaluation of how different formulations perform under biologically relevant conditions, including patient-specific variations in pH, enzymatic activity, and protein corona formation [83]. This workflow is well established in preclinical research and supports rational optimization of nanoparticle systems.

Artificial intelligence is increasingly used to accelerate nanoparticle design and optimization. Machine learning models can analyze relationships between material properties and biological outcomes, enabling prediction of drug loading efficiency, release kinetics, and cellular uptake [71,84]. In current practice, AI is most effective in reducing experimental search space and guiding formulation development rather than generating fully individualized therapies. For instance, models trained on nanoparticle libraries can recommend optimal size or surface chemistry for specific delivery goals, streamlining preclinical development [85].

The concept of adaptive dosing represents a further step toward personalization but remains at an earlier stage of translation. Clinically relevant decision points can already be identified, even if full automation is not yet realized. For example, treatment adjustments based on toxicity biomarkers—such as elevated liver enzymes during chemotherapy—can inform modifications to dosing regimens [86]. Imaging-based assessment of nanoparticle accumulation can also guide whether to maintain or modify a formulation strategy, rather than escalating dose indiscriminately [87]. In gene therapy, sequencing data identifying specific mutations (e.g., KRAS variants) can guide selection of siRNA or mRNA payloads delivered via polymeric nanoparticles [88].

Despite these advances, it is important to distinguish between what is currently feasible and what remains speculative. Biomarker-guided targeting, stimulus-responsive release systems, and AI-assisted formulation optimization are well established in preclinical studies, with some approaches advancing into early-phase clinical trials [71,73]. In contrast, fully integrated systems combining real-time biosensor feedback, AI-driven decision making, and dynamically adjustable nanoparticle dosing remain largely conceptual [26]. Similarly, routine clinical production of fully individualized nanoparticle formulations for each patient faces significant challenges related to manufacturing scalability, regulatory approval, and quality control [89].

In oncology, where tumor heterogeneity is a major barrier to effective treatment, these technologies hold particular promise. However, their near-term impact is likely to come from improved stratification and semi-personalized nanoparticle designs rather than fully autonomous adaptive systems. Continued progress will depend on the availability of high-quality, standardized datasets, advances in manufacturing reproducibility, and the development of regulatory frameworks capable of supporting AI-assisted therapeutics [88,89].

Tailoring polymeric nanoparticles using advanced characterization and AI represents a significant step toward personalized medicine, but its clinical realization is incremental rather than immediate. By grounding nanoparticle design in measurable patient-specific variables and clearly defining actionable clinical decision points, this approach can improve therapeutic efficacy and safety while maintaining a realistic translational trajectory.

## 4. Improvements in Drug Delivery and Manufacturing

Advances in drug delivery systems have significantly transformed modern therapeutics by enhancing drug efficacy, minimizing systemic toxicity, and enabling controlled and site-specific treatment. Traditional dosage forms often suffer from poor bioavailability, rapid systemic clearance, and non-specific distribution. The emergence of nanotechnology-based platforms—particularly polymeric nanoparticles, lipid nanoparticles, and nanofiber-based systems—has addressed many of these limitations. Key areas of development include advanced targeting and smart release systems, scalable and reproducible nanoparticle manufacturing, and nanofiber–nanoparticle hybrid materials for multifunctional biomedical applications.

### 4.1. Advanced Targeting and Release

#### 4.1.1. Passive and Active Targeting

**Figure 3:**
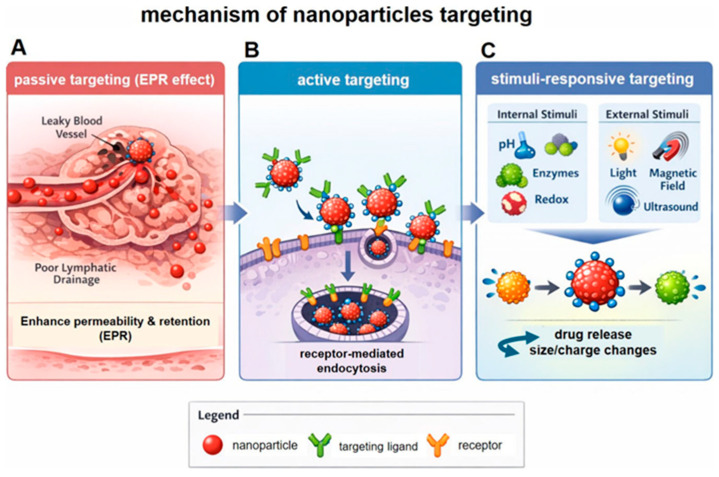
Difference between passive (**A**), active (**B**) and stimulus-responsive (**C**) targeting. Originally published in ref. [9] under CC BY 4.0 license. Copyright [2026] ACS Nano Medicine.

Nanoparticle-based drug delivery systems are designed to improve therapeutic outcomes by enhancing accumulation at diseased sites and increasing treatment specificity. Passive targeting (Figure 3) is primarily based on the EPR effect, in which nanomaterials tend to accumulate in solid tumors due to leaky and disorganized vasculature, increased vascular permeability, and impaired lymphatic drainage [85,86]. However, the EPR effect is highly heterogeneous and context-dependent. Its extent varies significantly across tumor types, anatomical locations, disease stages, and between preclinical tumor models and human cancers. Clinical studies have shown that nanoparticle accumulation in human tumors is often substantially lower and more variable than in animal models, which limits the predictability and general applicability of passive targeting strategies in clinical translation [57,58,59].

Active targeting (Figure 3) aims to improve cellular uptake and specificity by modifying nanoparticle surfaces with ligands such as antibodies, peptides, aptamers, or small molecules that bind to receptors overexpressed on target cells [90,91]. For example, folate receptor-targeted nanoparticles have shown improved cellular uptake in specific cancer models [92], while transferrin-functionalized nanoparticles have been extensively investigated to facilitate receptor-mediated transport across the blood–brain barrier (BBB) [93].

In the context of brain delivery, receptor-mediated transport systems have demonstrated enhanced nanoparticle uptake in preclinical studies; however, efficient and reproducible BBB penetration remains a major translational challenge. The BBB is a highly selective and dynamic interface, and its permeability changes in a heterogeneous and stage-dependent manner in neurodegenerative diseases such as Alzheimer’s disease. Although some disruption of BBB integrity has been reported in Alzheimer’s disease, it is neither uniform nor sufficient to guarantee effective drug delivery. Therefore, evidence supporting consistent nanoparticle transport across the BBB in both early and late stages of Alzheimer’s disease remains limited, and further validation in clinically relevant models is required before therapeutic efficacy can be confirmed [94,95,96].

Lipid nanoparticles (LNPs) are engineered to overcome major biological barriers that limit the therapeutic use of nucleic acids such as mRNA, siRNA, and gene-editing systems. These molecules are inherently unstable in physiological conditions and cannot readily cross cell membranes; therefore, LNPs are designed to protect the cargo, enhance cellular uptake, and enable controlled delivery to specific tissues [97,98].

LNPs are typically formed through rapid self-assembly of lipids and nucleic acids using methods such as microfluidic mixing. A lipid mixture—comprising ionizable lipids, phospholipids, cholesterol, and PEG lipids—is dissolved in ethanol and mixed with an aqueous solution containing the therapeutic cargo. The sudden change in solvent conditions drives spontaneous nanoparticle formation, encapsulating the nucleic acid within a stable lipid structure [99,100].

Recent advances in lipid nanoparticle engineering have introduced selective organ targeting strategies [92], where modifying lipid composition alters biodistribution profiles, enabling preferential delivery to the liver [93], spleen, or lungs [94]. Such approaches have broad implications for gene therapy and mRNA-based therapeutics.

#### 4.1.2. Stimulus-Responsive and Smart Release Systems

Smart drug delivery systems are designed to release therapeutic agents in response to internal or external stimuli. These systems enhance temporal and spatial control over drug release. Internal stimulus-responsive (Figure 3) systems exploit physiological differences between diseased and healthy tissues [5]. For instance, pH-sensitive nanoparticles release drugs in acidic tumor microenvironments or endosomal compartments [95]. Redox-responsive carriers respond to elevated intracellular glutathione levels. Enzyme-sensitive systems degrade in the presence of disease-specific enzymes [96]. pH-Responsive polymeric micelles and nanoparticles have demonstrated improved anticancer efficacy while minimizing systemic toxicity [97,101]. External stimulus-responsive systems include carriers activated by light, magnetic fields, ultrasound, or temperature changes [102]. Magnetic nanoparticles, for example, can be guided to tumor sites and heated via alternating magnetic fields to trigger drug release [103]. Light-responsive systems enable precise spatiotemporal drug activation in photodynamic therapy. Single-stimulus systems are generally simpler in design and have progressed further toward clinical translation, particularly pH- and enzyme-responsive platforms. However, their reliance on a single pathological cue limits specificity in heterogeneous disease microenvironments, such as solid tumors, where multiple overlapping signals (e.g., acidic pH, hypoxia, elevated ROS, and enzymatic activity) coexist with substantial spatial variability [104]. This multifactorial nature can exceed the selectivity of single-stimulus systems, potentially leading to premature drug release or off-target effects in healthy tissues that share similar conditions [105] (Table 4).

To overcome these limitations, multi-stimulus-responsive nanoparticles, particularly those based on block co-polymers, have been developed [106]. These advanced systems are engineered to respond to a combination of triggers—such as acidic pH, elevated reactive oxygen species, temperature changes, light exposure, and specific intracellular enzymes—thereby enhancing targeting precision [107]. Block co-polymers used in multi-stimulus-responsive nanoparticles are typically amphiphilic macromolecules composed of hydrophilic (e.g., PEG-poly(ethylene oxide (PEO)) and (bio)degradable or functional hydrophobic blocks (e.g., poly(lactic acid) (PLA), poly(lactic-*co*-glycolic acid) (PLGA), polycaprolactone (PCL), polyhistidine, or poly(β-amino esters)) [108]. These materials self-assemble in aqueous environments into micelles or core–shell nanoparticles and can be engineered with pH-, redox-, enzyme-, or temperature-sensitive linkages to enable controlled drug release in pathological microenvironments such as tumors or inflamed tissues [109].

They are mainly synthesized using controlled polymerization techniques, including ring-opening polymerization (for PLA-PLGA-based systems) and controlled radical polymerization methods such as RAFT (reversible addition–fragmentation chain transfer) and ATRP (atom transfer radical polymerization), which allow for the precise tuning of molecular weight, block architecture, and functional responsiveness. These design features are essential to achieving stable circulation and trigger-specific drug release in advanced nanomedicine applications [110,111]. As a result, they offer improved control over drug release across a wide range of biomedical applications, including cancer, diabetes, metabolic disorders, autoimmune diseases, neurological conditions, and bacterial infections [112]. In addition, biomimetic approaches, including cell membrane-coated nanoparticles, are being explored to further enhance biocompatibility with many organisms and targeting efficiency.

#### 4.1.3. pH-Sensitive Polymeric Nanoparticles

pH-sensitive polymeric nanoparticles are an important subclass of stimulus-responsive polymeric nanoparticles that have attracted considerable attention for controlled and targeted drug delivery [113,114]. These systems are specifically engineered to respond to variations in pH encountered in different physiological and pathological environments. In particular, the acidic microenvironment of tumors (pH ~6.5–6.9) [115], inflamed tissues, and intracellular organelles such as endosomes and lysosomes (pH ~4.5–6.7) provides a unique opportunity for selective drug release [116]. By exploiting these pH gradients, polymeric nanoparticles can enhance therapeutic efficacy while minimizing systemic toxicity.

The design of pH-sensitive polymeric nanoparticles relies primarily on the incorporation of ionizable functional groups or acid-labile linkages within the polymer backbone or side chains. Polymers containing weakly basic groups, such as amines, undergo protonation under acidic conditions, leading to increased hydrophilicity, electrostatic repulsion, and structural destabilization [117,118]. This often results in nanoparticle swelling or disassembly, thereby facilitating drug release. Conversely, polymers with acidic groups, such as carboxylic acids, may exhibit changes in ionization state that influence drug release kinetics and nanoparticle stability [119].

Another widely used strategy involves the incorporation of acid-cleavable bonds, including hydrazone, imine (Schiff base), acetal, and ketal linkages [120]. These bonds are stable at physiological pH (7.4) but undergo rapid hydrolysis in acidic environments [121]. When used to conjugate drugs to polymer chains or to stabilize nanoparticle structures, their cleavage enables site-specific release of therapeutic agents. This approach is particularly advantageous for delivering chemotherapeutic drugs, which often exhibit significant systemic toxicity when administered in free form.

Various polymeric systems have been developed to fabricate pH-sensitive nanoparticles, including linear polymers, block co-polymers, and crosslinked networks. Amphiphilic block co-polymers are among the most extensively studied materials, as they can self-assemble into core–shell structures such as polymeric micelles. In these systems, the hydrophobic core serves as a reservoir for poorly water-soluble drugs, while the hydrophilic shell ensures colloidal stability in biological fluids. Under acidic conditions, protonation of the polymer segments or cleavage of acid-labile bonds disrupts the core structure, resulting in rapid drug release.

(Bio)degradable polymers such as PLGA, poly(β-amino esters), and chitosan derivatives are frequently employed due to their biocompatibility with many organisms and regulatory approval potential. Chitosan, a naturally derived polysaccharide, is particularly attractive because of its inherent pH sensitivity arising from protonatable amino groups. At acidic pH, chitosan becomes positively charged and soluble, facilitating interaction with negatively charged cell membranes and enhancing cellular uptake. Similarly, poly(β-amino esters) exhibit a “proton sponge” effect, buffering endosomal pH and promoting endosomal escape through osmotic swelling and membrane disruption.

In cancer therapy, pH-sensitive polymeric nanoparticles offer several advantages [122]. They can passively accumulate in tumor tissues via the enhanced permeability and retention effect, which arises from the leaky vasculature and poor lymphatic drainage characteristic of tumors. Once localized, the acidic tumor microenvironment triggers drug release, increasing the local drug concentration while reducing systemic exposure. Furthermore, intracellular delivery is enhanced by the ability of these nanoparticles to escape endosomal compartments, thereby preventing lysosomal degradation of sensitive therapeutic agents such as proteins and nucleic acids.

Despite their considerable promise, several challenges continue to limit the clinical translation of pH-sensitive polymeric nanoparticles. Biological pH heterogeneity may compromise the precision and reproducibility of drug release, as significant variations can occur across patients, disease stages, and even among different regions of the same tumor. Additional barriers include nanoparticle instability during circulation, rapid clearance by the mononuclear phagocyte system, and the potential for immunogenicity. Surface modification strategies such as PEG conjugation are frequently employed to prolong circulation time and reduce immune recognition; however, excessive PEGylation may also impair cellular uptake and intracellular trafficking [122].

One of the most clinically investigated systems is MM-302, a HER2-targeted PEGylated liposomal doxorubicin conjugate. In a clinical trial, initial Phase I, dose-escalation study in patients with advanced HER2-positive breast cancer, MM-302 demonstrated measurable tumor uptake and acceptable safety, with imaging confirming intratumoral delivery of doxorubicin in metastatic lesions, including in sanctuary sites such as the brain. Although later Phase II studies showed mixed efficacy outcomes, MM-302 remains a benchmark example of antibody-directed, tumor-targeted liposomal anthracycline delivery and illustrates how ligand-directed polymeric nanoparticles can improve intratumoral accumulation while reducing systemic toxicity [123].

Another clinically relevant class includes thermosensitive and environment-responsive liposomal doxorubicin formulations, such as ThermoDox^®^ (lyso-thermosensitive liposomal doxorubicin). Early Phase I studies combining ThermoDox with localized hyperthermia or radiofrequency ablation demonstrated triggered intratumoral drug release and feasibility of external activation to enhance local chemotherapy deposition, particularly in liver malignancies [124]. These approaches highlight how physiological or externally modulated tumor environments can be leveraged to overcome limited passive pH gradients.

In parallel, polymeric micelle systems such as NC-6004 (nanoplatin) and related PEG–poly(glutamic acid) micelles have reached Phase I/II clinical trial evaluation of a new drug’s efficacy and safety in multiple solid tumors, including lung, biliary tract, and bladder cancers. NC-6004 demonstrated altered pharmacokinetics with reduced off-target toxicity and evidence of disease stabilization in a subset of patients, supporting the feasibility of polymer-based drug sequestration and tumor accumulation strategies in humans [125]. Similarly, PEGylated liposomal doxorubicin formulations continue to be clinically evaluated in combination regimens; for example, ongoing Phase II trials in metastatic triple-negative breast cancer are assessing PEGylated liposomal doxorubicin combined with endocrine modulators to improve response in difficult-to-treat disease subtypes [126].

More broadly, targeted immunoliposomes and antibody–drug-conjugated polymeric nanoparticles loaded with doxorubicin have entered Phase II clinical evaluation in advanced solid tumors, including triple-negative breast cancer, demonstrating the continued clinical translation of receptor-mediated tumor targeting strategies [127]. Collectively, these studies show that while fully pH-selective systems remain challenging to validate clinically, hybrid strategies combining targeting ligands, stimulus responsiveness (e.g., pH, heat, and enzymatic cleavage), and prolonged circulation are the dominant translational pathway.

Beyond oncology, pH-sensitive polymeric nanoparticles have demonstrated potential in the treatment of infectious and inflammatory diseases. Infected and inflamed tissues often exhibit reduced pH due to hypoxia and increased metabolic activity. Sun et al. developed pH-sensitive self-assembled polymer nanoparticles for targeted delivery of superoxide dismutase [128]. Zhao et al. fabricated pH-responsive, multifunctional methotrexate-loaded polymeric nanoparticles for the treatment of rheumatoid arthritis [129]. By designing nanoparticles that respond to these conditions, it is possible to achieve localized drug delivery and improved therapeutic outcomes. Additionally, these systems are being explored for oral drug delivery applications, where they can protect drugs from degradation in the acidic gastric environment and enable controlled release in the intestine.

Despite their promising attributes, several challenges must be addressed for the successful clinical translation of pH-sensitive polymeric nanoparticles. The heterogeneity of pH in biological systems can affect the precision of drug release. Variability between patients, disease stages, and even within different regions of a single tumor may lead to inconsistent therapeutic outcomes. Moreover, issues related to nanoparticle stability in circulation, rapid clearance by the mononuclear phagocyte system, and potential immunogenicity remain significant barriers. pH-Sensitive polymeric nanoparticles represent a highly promising platform for targeted and controlled drug delivery [118,130].

#### 4.1.4. Redox-Responsive Polymer Nanoparticles

Redox-responsive polymer nanoparticles have emerged as a promising class of smart drug delivery systems due to their ability to exploit the distinct redox gradients between physiological and pathological environments [131]. In particular, intracellular compartments such as the cytosol and cell nucleus exhibit significantly higher concentrations of reducing agents, notably glutathione, compared with extracellular fluids. This difference provides a biochemical trigger that can be harnessed to achieve controlled and site-specific drug release.

Polymer nanoparticles designed for redox responsiveness typically incorporate chemical linkages that are stable under normal physiological conditions but cleavable in reducing environments. Among these, disulfide bonds (–S–S–) are the most widely used due to their reversible cleavage in the presence of thiols like glutathione [132,133]. When nanoparticles containing disulfide linkages enter cells, the elevated intracellular glutathione concentration (2–10 mM) reduces the disulfide bonds into thiols, leading to structural destabilization of the carrier and subsequent release of the encapsulated therapeutic payload.

Various polymer architectures have been developed to construct redox-sensitive nanoparticles, including block co-polymers, micelles, dendrimers, and nanogels [134,135]. Amphiphilic block co-polymers are particularly attractive because they can self-assemble into core–shell nanostructures in aqueous environments. In such systems, hydrophobic drug molecules are typically loaded into the core, while the hydrophilic shell ensures colloidal stability and prolongs circulation time. By incorporating disulfide bonds either in the polymer backbone, crosslinkers, or at the interface between hydrophilic and hydrophobic segments, these nanoparticles can undergo rapid disassembly in reductive intracellular environments. One example is the advanced PEG-disulfide-PLGA nanoparticles (PEG-SS-PLGA) having redox-dependent disulfide functional groups in their structure. Recent preclinical studies indicate the potential to increase therapeutic efficacy, reduce multidrug resistance, and provide theranostic imaging capabilities using these nanoparticles [136].

Nanogels represent another versatile platform for redox-responsive delivery. These highly crosslinked, water-swollen polymer networks can encapsulate a wide range of therapeutic agents, including small molecules, proteins, and nucleic acids.

In cancer therapy, redox-responsive polymer nanoparticles offer significant advantages. Tumor tissues often exhibit elevated levels of glutathione compared with normal tissues, further enhancing the selectivity of redox-triggered systems [137]. Commonly used polymers in nanoparticle production include PEG, PLGA, and poly(amino acids), modified to include fragments sensitive to redox reactions [138]. Liu et al. developed a strategy to prepare PEGylated, redox-responsive nanostructures for efficient loading and delivery of doxorubicin (DOX) [139]. Despite their potential, several challenges remain in the clinical translation of redox-responsive polymer nanoparticles. One major concern is the variability in redox conditions across different cell types and disease states, which may affect the predictability of drug release. Furthermore, premature degradation due to extracellular thiols or instability during storage can compromise efficacy. Strategies to address these issues include optimizing polymer composition, increasing steric protection around sensitive linkages, and incorporating additional stabilizing interactions. Redox-responsive polymer nanoparticles represent a highly adaptable and effective platform for controlled drug delivery. By leveraging the intrinsic redox differences between extracellular and intracellular environments, these systems enable precise spatiotemporal release of therapeutics. Recent studies highlight that disulfide-based polymeric nanoparticles can significantly enhance intracellular drug accumulation while minimizing off-target toxicity; however, variability in tumor glutathione (GSH) levels and heterogeneous microenvironmental conditions may affect release kinetics and therapeutic performance. Consequently, while numerous redox-sensitive platforms demonstrate strong preclinical efficacy, translation into clinically robust systems remains limited and requires further validation under physiologically relevant conditions and in human settings [140].

#### 4.1.5. Enzyme-Sensitive Polymeric Nanoparticles

Enzyme-sensitive systems represent a powerful strategy for the controlled degradation of polymeric nanoparticles, enabling targeted drug delivery and improved therapeutic outcomes [141,142]. These systems exploit the aberrant overexpression or elevated activity of specific enzymes in pathological environments such as tumors, inflamed tissues, and infection sites to trigger the selective breakdown of polymer matrices and the subsequent release of encapsulated cargo in a spatially and temporally controlled manner [141,142,143].

A major advantage of these systems is their ability to achieve site-specific cargo release while minimizing premature leakage during systemic circulation. This is accomplished by incorporating enzyme-cleavable motifs within the polymer backbone, crosslinkers, or surface coatings that are specifically recognized by target enzymes.

The fundamental mechanism underlying enzyme-responsive nanoparticles is based on enzyme–substrate recognition followed by catalytic bond cleavage, rather than nonspecific chemical degradation. Enzymes selectively bind to short peptide sequences, polysaccharide motifs, or synthetic labile bonds integrated into the nanoparticle architecture. Upon binding, catalytic hydrolysis or bond scission occurs, targeting peptide, glycosidic, or ester linkages. This enzymatic action progressively destabilizes the nanoparticle structure, ultimately leading to disassembly and controlled drug release.

A major design challenge is achieving an optimal balance between systemic stability and enzyme-triggered responsiveness, ensuring that nanoparticles remain stable during circulation but rapidly degrade in enzyme-rich pathological microenvironments. This requires the careful tuning of substrate specificity, enzyme affinity, bond cleavage kinetics, and polymer architecture.

Among the most widely studied systems are protease-responsive nanoparticles, particularly those sensitive to matrix metalloproteinases (MMP-2—Gelatinase A, 72 kDa; MMP-9—Gelatinase B, 92 kDa) and cathepsin B, which are frequently upregulated in tumor progression and metastasis [144,145,146,147]. Incorporation of MMP-cleavable peptide sequences into PEG–PLGA or liposomal systems enables selective degradation within the tumor extracellular matrix, thereby enhancing intratumoral penetration and therapeutic efficacy. Similarly, cathepsin B-sensitive linkers facilitate intracellular activation following endocytosis and lysosomal trafficking.

In parallel, polysaccharide-based enzyme-responsive systems have attracted significant attention. Natural polymers such as hyaluronic acid, dextran, and chitosan can be selectively degraded by hyaluronidase, dextranase, and lysozyme, respectively [148,149,150,151,152]. Notably, hyaluronic acid-based nanoparticles offer dual functionality by enabling CD44 cell surface adhesion receptor-mediated targeting and enzymatic degradation within the tumor microenvironment, thereby integrating active targeting with stimulus-responsive drug release.

Additionally, synthetic polymer systems incorporating enzyme-labile ester or peptide-like bonds have been developed to enhance degradation specificity. For example, modified PLGA systems containing enzyme-sensitive linkers exhibit accelerated degradation in pathological enzymatic environments compared with passive hydrolysis alone [153,154].

Overall, the design of enzyme-responsive nanoparticles requires careful consideration of enzyme specificity, catalytic kinetics, and expression variability. The cleavage rate must be sufficiently rapid to achieve therapeutic efficacy, yet sufficiently controlled to maintain stability during circulation. Moreover, interpatient variability in enzyme expression and activity may significantly influence therapeutic performance, highlighting the importance of personalized design strategies.

Although enzyme-responsive nanoparticles offer significant promise for targeted therapy, their clinical translation remains challenged by enzyme heterogeneity, kinetic variability, and potential off-target enzymatic activity. Nevertheless, by leveraging disease-associated enzymatic signatures, these systems provide a robust platform for enhancing therapeutic efficacy while reducing systemic toxicity. Table 5 summarizes the used enzyme-responsive polymeric nanoparticle systems.

### 4.2. Scalable and Reproducible Manufacturing

Despite promising laboratory findings, the clinical translation of nanomedicine depends heavily on the development of robust, scalable, and reproducible manufacturing processes. Nanoparticles are highly sensitive systems in which small variations in formulation or processing conditions can significantly alter physicochemical properties such as particle size, dispersity index, surface charge (zeta potential), drug loading efficiency, and stability [149]. These attributes directly influence biodistribution, pharmacokinetics, cellular uptake, therapeutic efficacy, and safety. Consequently, ensuring consistency during large-scale production is a central requirement for successful commercialization.

A key strategy to address these challenges is the adoption of quality-by-design (QbD) principles, which emphasize predefined quality targets, systematic risk assessment, and a thorough understanding of process–product relationships. Within this framework, critical quality attributes (CQAs) and critical process parameters are identified early in development, enabling more predictable scale-up and reducing late-stage variability.

One of the most critical challenges in nanoparticle manufacturing is batch-to-batch variability [150]. In laboratory-scale production, synthesis is typically performed under tightly controlled conditions and small volumes. However, when scaling up to pilot or industrial levels, maintaining identical mixing dynamics, solvent diffusion rates, shear forces, and temperature control becomes significantly more complex [151]. Even minor differences in these parameters can lead to substantial variations in nanoparticle size distribution and surface properties.

To overcome these limitations, continuous processing approaches are increasingly being explored as an alternative to conventional batch manufacturing. Continuous flow systems enable more uniform energy input, improved reproducibility, and better control over nucleation and growth kinetics. In particular, microfluidic-based synthesis and microfluidic scale-up strategies allow for precise control of mixing at the microscale, producing highly uniform nanoparticles with narrow size distributions and enhanced reproducibility across production runs.

In parallel, the integration of process analytical technology (PAT) has become essential to the real-time monitoring and control of nanoparticle synthesis. PAT tools such as dynamic light scattering probes, UV–Vis spectroscopy, Raman spectroscopy, and in-line particle sizing systems enable continuous feedback on critical quality attributes during production. This real-time data acquisition supports adaptive process control, reduces variability, and enhances compliance with regulatory expectations for consistent product quality.

Since regulatory agencies require strict consistency in product quality, such variability poses serious obstacles to compliance with good manufacturing practice (GMP) standards [152]. Therefore, combining QbD-driven design with continuous manufacturing and PAT-enabled control represents a powerful framework for improving scalability and reproducibility.

Aggregation during storage represents another major limitation [151]. Nanoparticles are thermodynamically unstable and prone to aggregation due to van der Waals and surface interactions [152]. Aggregation can increase particle size, alter drug release profiles, reduce bioavailability, and potentially trigger immunogenic responses [153]. Environmental factors such as pH, ionic strength, and temperature significantly affect colloidal stability [154]. To mitigate this, formulation strategies often incorporate stabilizers, surfactants, or polymer coatings such as PEG to improve steric stabilization [6]. Lyophilization is frequently employed to enhance shelf life; however, improper freeze-drying protocols may cause structural collapse or loss of drug integrity.

Another significant challenge is sterilization without compromising functionality. Clinical-grade nanoparticle formulations must meet stringent sterility requirements [170]. Common sterilization methods include sterile filtration, autoclaving, ionizing and non-ionizing radiation, and chemical treatments; however, these techniques may alter nanoparticle properties depending on composition and treatment conditions [171]. Consequently, selecting the appropriate sterilization method requires balancing sterility assurance with preservation of structural and functional integrity.

High production costs further complicate scale-up. Manufacturing nanomedicines requires specialized equipment such as high-pressure homogenizers, microfluidic systems [172,173], and spray-drying units. Transitioning from batch production to continuous manufacturing platforms, although capital-intensive, can significantly reduce variability and long-term production costs while improving scalability [174]. Additionally, the implementation of QbD and PAT can reduce waste, re-processing, and failed batches, further improving cost efficiency.

Finally, regulatory compliance remains a major barrier [175]. Regulatory authorities demand comprehensive characterization of nanoparticle physicochemical properties, stability data, toxicity assessments, and validated production protocols [176]. The use of QbD frameworks, combined with continuous manufacturing and PAT, is increasingly viewed as aligned with regulatory expectations because it provides enhanced process understanding and traceability. Nevertheless, any modification in formulation or processing conditions may still require additional validation, contributing to development complexity and slowing clinical translation.

Scalable and reproducible nanoparticle manufacturing therefore requires an integrated strategy combining stringent control of processing parameters, continuous and microfluidic-enabled manufacturing technologies, real-time process analytical technology, quality-by-design principles, advanced characterization methods, cost-effective production strategies, and adherence to evolving regulatory standards. Addressing these challenges is essential to bridging the gap between laboratory innovation and clinical application in nanomedicine.

### 4.3. Nanofiber–Nanoparticle Hybrids

Polymer nanoparticle-reinforced nanofiber systems can be fabricated through three principal pathways: (i) the incorporation of nanoparticles as fillers within composite fibers, (ii) the post-fabrication coating of fiber surfaces, and (iii) the dispersion of nanoparticles within the electrospinning dope followed by in situ integration during fiber formation. Among these, direct incorporation into the spinning solution is the most widely applied approach, as it enables relatively uniform distribution of nanoparticles throughout the fiber matrix during electrospinning. In this configuration, nanoparticles become physically embedded within the polymeric nanofiber core, resulting in true nanocomposite fibers with bulk-level reinforcement effects [177]. In contrast, surface coating strategies localize nanoparticles predominantly at the fiber–environment interface, which is advantageous for surface-driven functions such as antibacterial activity or catalytic performance but contributes less to mechanical reinforcement [178]. In situ incorporation during electrospinning represents a hybrid approach in which nanoparticle distribution is governed by jet dynamics, solvent evaporation, and interfacial interactions, often resulting in either homogeneous dispersion or partial migration toward the fiber surface, depending on processing conditions [179].

The main problem in all manufacturing methods is achieving stable dispersion of nanoparticles in the polymer matrix before and during the electrospinning process. Nanoparticle aggregation is a critical issue, as it leads to non-uniform fiber morphology, nozzle clogging, and localized stress concentration sites that negatively affect performance [180]. To mitigate aggregation, several strategies are commonly employed, including surface functionalization of nanoparticles to enhance compatibility with the host polymer, the use of surfactants or steric stabilizers, and intensive pre-processing techniques such as ultrasonication or high-shear mixing. In addition, careful selection of solvent systems is essential to ensuring simultaneous compatibility of both polymer and nanoparticles while maintaining appropriate solution conductivity and viscosity for stable jet formation [181]. In many cases, controlling nanoparticle concentration below a critical loading threshold is necessary to prevent percolation-driven agglomeration unless a continuous nanoparticle network is intentionally desired.

The incorporation of polymer nanoparticles significantly influences the mechanical behavior of electrospun fibers. When nanoparticles are homogeneously dispersed and exhibit strong interfacial adhesion with the polymer matrix, they act as effective nanoscale reinforcements, improving stress transfer and increasing tensile strength, stiffness (Young’s modulus), and fatigue resistance [182]. This reinforcement effect is attributed to restricted polymer chain mobility and efficient load transfer across the nanoparticle–polymer interface. In addition, depending on nanoparticle deformability, toughness and elongation at break may also be enhanced through energy dissipation mechanisms such as localized plastic deformation or shear yielding. However, poor dispersion or nanoparticle aggregation has the opposite effect, introducing defects that act as crack initiation sites and significantly reducing mechanical performance. Therefore, interfacial compatibility and dispersion quality are decisive factors governing the final mechanical properties of nanofiber–nanoparticle hybrid systems [183].

Drug release from pure nanofibers is typically governed by diffusion through the polymer matrix and by polymer degradation, which may result in either burst release (if drugs are near the surface) or slow, sustained release (for hydrophobic matrices). In contrast, nanoparticle-loaded fibers enable multi-phase release mechanisms, including nanoparticle diffusion, matrix swelling, and barrier-controlled transport. This often results in more programmable release profiles, including reduced initial burst and extended delivery duration [184,185]. For example, core–shell or nanoparticle-embedded fibers can introduce additional diffusion barriers, significantly prolonging release compared with monolithic nanofibers. Conversely, poorly stabilized nanoparticle dispersions may accelerate release unpredictably due to aggregation-induced defects or polymer discontinuities.

Nanofiber–nanoparticle hybrid systems (NNHs) represent a multifunctional class of biomaterials that combine the structural advantages of electrospun polymer nanofibers with the tunable physicochemical properties of nanoparticles. Nanofibers provide high surface area, extracellular matrix (ECM)-like morphology, and favorable cell-interactive properties but are often limited in therapeutic functionality. Nanoparticle incorporation overcomes this limitation by enabling controlled drug delivery, antimicrobial activity, imaging capability, and stimulus responsiveness, thereby bridging the gap between structural scaffolds and active therapeutic systems [186,187].

This structural–functional coupling is particularly important in drug delivery. For example, electrospun PCL-based systems containing protein or drug-loaded nanoparticles can achieve prolonged and dual-phase release, combining an initial burst with sustained diffusion over days to weeks [188]. More advanced core–shell architectures further refine this behavior by introducing diffusion barriers that slow release kinetics and improve dose stability. Similarly, incorporation of stimulus-responsive nanoparticles (e.g., photothermal or magnetic systems) enables externally triggered release, improving spatial and temporal control of therapy [189,190].

In tissue engineering, NNHs function as bioactive ECM mimics where polymer matrices (e.g., PCL, PLA, gelatin, and chitosan) provide mechanical support, while nanoparticles enhance biological signaling. This synergy improves cell adhesion, proliferation, and differentiation while also enabling antibacterial protection and mechanical reinforcement [191,192,193,194]. In antimicrobial applications, metal or metal oxide nanoparticles such as silver (Ag), zinc oxide (ZnO), and titanium dioxide (TiO_2_) enhance bacterial inactivation through reactive oxygen species generation and membrane disruption, while the nanofibrous architecture increases contact efficiency due to its high surface-area-to-volume ratio [195].

A major distinction of NNHs compared with standalone nanoparticles or nanofibers is their hierarchical functional integration: nanoparticles alone suffer from aggregation, rapid clearance, and poor localization, while nanofibers alone lack active functionality. Hybrid systems resolve this by embedding or immobilizing nanoparticles within a mechanically supportive scaffold that regulates exposure, stability, and release behavior. However, this integration introduces critical design constraints, particularly in biomedical contexts. Despite their advantages, hybrid systems raise important biosafety concerns, particularly when inorganic nanoparticles such as Ag, ZnO, or TiO_2_ are used. Potential issues include nanoparticle leaching from the fiber matrix, accumulation in biological tissues, and long-term cytotoxicity due to oxidative stress or ion release. Even when nanoparticles are embedded within fibers, incomplete encapsulation or polymer degradation can lead to gradual release into surrounding media [196,197]. Therefore, careful consideration of nanoparticle type, surface chemistry, and encapsulation stability is essential, especially for biomedical applications. Strategies such as core–shell architectures, covalent immobilization, and biodegradable but with controlled-degradation polymers are commonly used to mitigate these risks.

## 5. Broader Applications and Safety

### 5.1. Tissue Engineering and Regenerative Medicine

Polymer nanoparticles have become a versatile platform in tissue engineering and cell-related applications, particularly for targeted therapies and the fabrication of biocompatible scaffolds for various biological systems. Advances in nanotechnology and biomaterials science have enabled the development of multifunctional polymer-based systems that closely mimic the native ECM, improving tissue regeneration outcomes. Polymeric nanoparticles exhibit tunable physicochemical properties such as size, surface charge, porosity, and degradation rate, allowing for precise control of drug delivery and cellular interactions. Furthermore, stimulus-responsive polymeric nanoparticles (e.g., pH-, temperature-, or enzyme-sensitive) enable controlled release in specific microenvironments, improving therapeutic efficacy in tissue repair and regeneration [198]. In tissue engineering, polymer scaffolds serve as 3D templates that support cell adhesion, proliferation, migration, and differentiation. However, conventional polymer scaffolds often have limited mechanical strength and bioactivity. To address these limitations, recent research has focused on incorporating nanoparticles into polymer matrices to form nanocomposite scaffolds. For example, the addition of metallic, ceramic or polymeric nanoparticles increases mechanical strength, antibacterial properties and cellular responses such as adhesion and differentiation. Moreover, nanoparticles increase the surface area and provide nanoscale topographical cues that mimic the natural ECM, promoting more efficient tissue regeneration.

Applications of polymer nanoparticle-based scaffolds include many tissue types, including bone, cartilage, skin, nervous, and cardiovascular tissues [199,200,201]. In wound healing applications, injectable chitosan/alginate hydrogels loaded with Ag or ZnO nanoparticles have demonstrated accelerated closure of full-thickness infected wounds in murine and porcine skin models. These scaffolds exhibit sustained antimicrobial activity while simultaneously promoting granulation tissue formation and re-epithelialization through vascular endothelial growth factor (VEGF)-mediated angiogenic signaling [202]. A clinically relevant advancement includes electrospun nanofibrous dressings (e.g., PCL/gelatin systems) incorporating antibiotic-loaded polymeric nanoparticles, which have been evaluated in diabetic wound models and shown to significantly reduce bacterial burden while enhancing neovascularization and collagen deposition [203].

In neural tissue engineering, conductive polymer-based nanocomposites such as polypyrrole/collagen or poly(3,4-ethylenedioxythiophene):poly(styrene sulfonic acid) (PEDOT:PSS)-functionalized fibrous scaffolds have been tested in rat sciatic nerve injury models. These scaffolds support aligned axonal regeneration and functional recovery by providing both topographical guidance and electrical conductivity that mimics endogenous neural signaling environments [204]. Clinically relevant nerve guidance conduits based on PLGA or PCL combined with nanoparticles (e.g., graphene oxide or gold nanoparticles) are currently under preclinical evaluation for peripheral nerve repair, demonstrating improved Schwann cell migration and myelination compared with nonfunctionalized conduits [205].

In cardiovascular applications, decellularized extracellular matrix (dECM) scaffolds functionalized with polymer nanoparticles have been explored for vascular graft engineering. For example, PLGA nanoparticle-loaded electrospun vascular grafts have been implanted in rat abdominal aorta models, showing reduced thrombogenicity and improved endothelialization compared with conventional synthetic grafts [206]. Similarly, elastomeric polyurethane scaffolds incorporating nitric oxide (NO)-releasing nanoparticles have demonstrated improved vasodilation and anti-inflammatory responses in vivo, supporting their potential for small-diameter vascular graft applications [207]. These innovations enable the design of intelligent scaffolds. These are capable of dynamically interacting with cells. They can also adapt to physiological conditions.

### 5.2. Drug Overdose Mitigation

The development of nanoparticle-based nanosponges represents a promising strategy in nanomedicine for mitigating drug toxicity by physically removing excess therapeutic agents from the bloodstream. Nanosponges are nanoscale, porous, 3D polymeric structures—often synthesized from crosslinked cyclodextrins or similar materials—capable of encapsulating or adsorbing a wide range of molecules, including both hydrophilic and hydrophobic drugs [208]. Their high surface area and tunable chemistry enable the selective binding of toxic compounds, making them particularly attractive for overdose management and detoxification applications.

Mechanistically, nanosponges function through adsorption and sequestration. Once administered, typically via intravenous injection, they circulate through the bloodstream and bind free drug molecules through non-covalent interactions such as hydrophobic forces, van der Waals interactions, or inclusion complexation. This reduces the bioavailable concentration of the drug, thereby limiting its interaction with healthy tissues and decreasing systemic toxicity. The nanosponge–drug complex is subsequently cleared by the reticuloendothelial system, primarily in the liver and spleen. This approach contrasts with traditional antidotes, which often rely on biochemical antagonism rather than physical removal.

Recent research highlights several nanosponge designs. Cyclodextrin-based nanosponges are among the most studied due to their biocompatibility with many organisms and ability to form inclusion complexes with drug molecules [209,210,211]. Biomimetic nanosponges, such as those coated with red blood cell membranes, act as decoys for toxins, binding them before they interact with native cells. Additionally, advanced functionalized nanosponges can be engineered with targeting ligands or stimulus-responsive release systems, further enhancing specificity and safety [212,213].

The clinical potential of nanosponges spans multiple applications, including chemotherapy detoxification, treatment of drug overdoses, and neutralization of bacterial toxins. For instance, they may reduce adverse effects associated with potent drugs by maintaining therapeutic concentrations while removing excess doses [214]. Studies also emphasize their ability to improve drug stability, solubility, and controlled release, thereby enhancing therapeutic efficacy while minimizing side effects. Table 6 summarizes recent nanosponge applications.

Despite these advantages, several challenges remain. These include ensuring precise selectivity to avoid removing beneficial drug levels, achieving scalable and reproducible synthesis, and addressing regulatory and safety concerns. Furthermore, long-term biocompatibility with many organisms and clearance pathways must be fully understood before widespread clinical adoption.

Nanosponge nanoparticles represent a promising and potentially transformative strategy for managing drug toxicity by physically sequestering excess therapeutic agents from the bloodstream [218]. However, it is important to distinguish clearly among drug sequestration, detoxification, and targeted drug delivery, as these processes involve different mechanisms and therapeutic goals. While nanosponges are primarily designed to adsorb and remove circulating compounds, their role in controlled drug delivery depends on additional functionalization and release mechanisms [219,220].

Despite their advantages, several challenges and risks must be more explicitly considered. A key limitation is the potential lack of selectivity, as nanosponges may inadvertently bind and remove therapeutically beneficial drug concentrations, potentially reducing efficacy or causing subtherapeutic exposure. This risk is particularly relevant in treatments requiring tight pharmacokinetic control, such as chemotherapy. Additionally, issues related to scalable synthesis, batch reproducibility, and regulatory approval remain significant barriers to clinical translation. Long-term biocompatibility with many organisms, biodistribution, and clearance pathways also require comprehensive evaluation to ensure safety across diverse patient populations. Overall, while nanosponge-based systems offer a novel approach that combines detoxification with elements of drug modulation, their clinical success will depend on achieving a careful balance between effective toxin removal and preservation of therapeutic drug levels. Continued advancements in material engineering, coupled with rigorous clinical validation, will be essential to translating these systems from experimental platforms to safe and effective medical interventions.

### 5.3. Sustainable and Eco-Friendly Materials

(Bio)degradable polymers have emerged as a promising strategy to reduce the environmental and biological burden of nanomedicine, particularly in addressing concerns related to persistence, toxicity, and accumulation of nanomaterials. In recent years, research has increasingly focused on designing polymeric nanoparticles that degrade into non-toxic by-products while maintaining therapeutic efficacy. Importantly, many natural polymers such as chitosan, cellulose, starch, and zein are inherently biodegradable, exhibit high biocompatibility with many organisms, and are generally recognized as safe (GRAS) by the U.S. Food and Drug Administration (FDA), supporting their longstanding use in biomedical and food-related applications [221,222,223].

Nanomedicine relies heavily on nanoscale carriers for drug delivery, imaging, and diagnostics. However, conventional non-degradable nanomaterials may accumulate in biological systems and the environment, raising long-term safety concerns. (Bio)degradable polymers such as PLA, PLGA, and PCL [224], along with natural polymers, have therefore been widely investigated as safer alternatives. PLGA, for instance, degrades under physiological conditions into lactic acid and glycolic acid, which are metabolized through endogenous pathways, thereby reducing systemic toxicity and improving clearance profiles [225,226].

A critical conceptual distinction must be made between degradation in vivo and environmental degradability after disposal, as these processes are fundamentally different. In vivo degradation occurs under tightly regulated physiological conditions driven by hydrolysis and enzymatic activity, enabling predictable breakdown and elimination from the body. In contrast, environmental degradation depends on external and highly variable conditions such as temperature, microbial activity, oxygen availability, and UV exposure. Consequently, a polymer that is safely degradable in the human body may not fully mineralize in natural ecosystems, and vice versa. Partial degradation and fragmentation into micro- or nanoscale residues may still occur in the environment, even for biodegradable polymers, highlighting the importance of designing materials with complete and predictable degradation pathways across both contexts [227,228,229].

A major advantage of (bio)degradable polymeric nanoparticles is their tunable degradation rate. By modifying molecular weight, co-polymer ratio, and crystallinity or incorporating environmental triggers (e.g., pH or enzymatic sensitivity), researchers can control drug release profiles and ensure that degradation is primarily localized at the target site. This reduces off-target accumulation and minimizes unintended environmental release following excretion. Additionally, (bio)degradable systems generally demonstrate improved biocompatibility with many organisms and reduced immunogenicity compared with many inorganic nanomaterials [230,231,232].

From a translational and lifecycle perspective, (bio)degradable polymers should be understood not only as safer biomedical carriers but also as materials embedded within a full design–use–disposal continuum. Their sustainability profile depends on both clinical performance and post-use fate. Lifecycle assessment (LCA) has therefore become an essential tool for evaluating their true environmental benefit, incorporating raw material sourcing, synthesis energy demand, manufacturing impact, and end-of-life degradation behavior [229,233]. While bio-based polymers reduce reliance on fossil-derived feedstocks, their overall sustainability is strongly influenced by waste management infrastructure and real-world degradation conditions. Finally, the development of (bio)degradable polymer-based nanomedicine aligns strongly with the United Nations Sustainable Development Goals (SDGs). It directly contributes to SDG 3 (Good Health and Well-being) by enabling safer and more biocompatible therapeutic systems, SDG 12 (Responsible Consumption and Production) by promoting reduced material persistence and improved lifecycle design, and SDG 13 (Climate Action) by lowering dependence on fossil-based polymers and mitigating long-term environmental pollution. Collectively, these systems illustrate how sustainable materials engineering can support both clinical innovation and environmental stewardship, ensuring that advances in nanomedicine are translated responsibly across biological and ecological boundaries [234].

## 6. Current and Future Directions—Final Conclusions

The rapid evolution of polymer-based nanoparticle systems is redefining the future of drug delivery and therapeutic precision. As highlighted throughout this manuscript, advances in nanocarrier engineering have enabled highly controlled, targeted, and multifunctional platforms that address many shortcomings of conventional therapies. These systems offer improved bioavailability, reduced systemic toxicity, and the ability to deliver therapeutics in a spatially and temporally controlled manner. Such capabilities are particularly valuable in the treatment of complex and chronic diseases, where traditional approaches often fail to achieve optimal outcomes.

A central theme emerging from current research is the shift toward personalization. The integration of nanotechnology with molecular diagnostics and AI is paving the way for patient-specific therapeutic strategies. Polymer nanoparticles can now be engineered based on individual biological signatures, including genetic, proteomic, and metabolic profiles. This level of customization enables a more precise targeting of diseased tissues while minimizing off-target effects. Furthermore, adaptive dosing systems—supported by biosensors and real-time monitoring—introduce a dynamic dimension to treatment, allowing therapies to respond continuously to physiological changes. Theranostic platforms represent another significant advancement in this field. By combining diagnostic and therapeutic functionalities within a single nanoparticle system, clinicians can simultaneously monitor disease progression and treatment response. This dual capability enhances early detection, improves treatment accuracy, and reduces the need for invasive procedures. When integrated with advanced imaging technologies and data-driven models, theranostic nanoparticles have the potential to significantly improve clinical decision making and patient outcomes.

Despite these promising developments, several challenges must be addressed before polymer nanoparticle systems can achieve widespread clinical implementation. One of the primary obstacles lies in large-scale manufacturing and reproducibility. While laboratory-scale synthesis allows for precise control over nanoparticle properties, translating these processes into industrial production remains complex. Variability in size, surface characteristics, and drug loading efficiency can impact both safety and efficacy. Advances in microfluidic technologies and automated manufacturing systems offer potential solutions by enabling more consistent and scalable production methods.

Regulatory considerations also play a critical role in the translation of nanomedicine from research to clinical practice. The unique properties of polymer nanoparticles—such as their size, surface functionality, and (bio)degradability—pose challenges for existing regulatory frameworks, which are often not fully equipped to evaluate nanoscale materials. Clear guidelines for characterization, safety assessment, and long-term toxicity studies are essential to ensuring patient safety and facilitating approval processes. The development of standardized assessment protocols will require collaboration between scientists, industry representatives and regulatory bodies.

Ethical considerations must also be taken into account, particularly in the context of personalized nanomedicine. The use of patient-specific data, including genomic information, raises important questions regarding data privacy, security, and equitable access to advanced therapies. Ensuring that these innovations are accessible across diverse populations, rather than limited to well-resourced healthcare systems, will be crucial to preventing disparities in care.

Sustainability is emerging as an additional priority in the development of polymer nanoparticles. Traditional nanomaterials often rely on non-degradable, petroleum-based polymers that contribute to environmental burden. The transition toward (bio)degradable and renewable polymer systems represents a significant step forward in aligning nanomedicine with global sustainability goals. These materials not only reduce environmental impact but also improve biocompatibility with many organisms and reduce long-term accumulation within the body. Looking ahead, the convergence of disciplines—including materials science, bioengineering, data science, and clinical medicine—will drive continued innovation in this field. Artificial intelligence is expected to play an increasingly important role in nanoparticle design, optimizing parameters such as size, shape, surface chemistry, and drug release profiles. Similarly, advances in biosensor technology will enhance the development of responsive systems capable of real-time therapeutic adjustments.

Polymer nanoparticles are positioned at the forefront of next-generation medical technologies. Their ability to support targeted, adaptive, and personalized therapies marks a transformative shift in healthcare. While technical, regulatory, and ethical challenges remain, ongoing interdisciplinary efforts and technological advancements are steadily bridging the gap between experimental research and clinical application.

Several platforms—particularly those involving PEGylated carriers, polymer–drug conjugates, and stimulus-responsive delivery systems—are now the closest to clinical translation, a development facilitated by greater reproducibility of results, scalable synthesis approaches, and growing regulatory familiarity. In contrast, more complex architectures, including fully adaptive theranostic systems and AI-integrated personalized nanomedicine, remain largely exploratory, with proof-of-concept success but limited clinical validation to date.

A critical change in the field is the movement from capability-driven design toward clinically constrained engineering. Rather than maximizing multifunctionality, emerging efforts increasingly prioritize robustness, manufacturability, and safety—criteria that ultimately determine translational success. This transition reflects a broader maturation of nanomedicine, where performance in controlled laboratory settings must align with real-world clinical and regulatory requirements.

Despite significant progress, the path to widespread clinical implementation remains uneven. To accelerate translation while maintaining safety and efficacy, the field must focus on a set of clearly defined and prioritized challenges:

(i) Clinical validation and standardization of efficacy endpoints. While preclinical data are abundant, there is a critical need for well-designed clinical studies that demonstrate clear therapeutic advantage over existing treatments. Harmonized endpoints and better patient stratification strategies will be essential.

(ii) Scalable and reproducible GMP manufacturing. Batch-to-batch variability in nanoparticle size, surface properties, and drug loading continues to limit industrial translation. Robust, scalable manufacturing platforms—particularly those leveraging microfluidics and automation—must be further developed and standardized.

(iii) Long-term safety, biodistribution, and clearance. A comprehensive understanding of nanoparticle fate in vivo remains incomplete. Long-term toxicity, immunogenicity, and accumulation—especially for non-fully degradable systems—require systematic investigation across diverse patient populations.

(iv) Regulatory framework alignment and standardization. Existing regulatory pathways are not fully adapted to nanoscale therapeutics. Clear, globally harmonized guidelines for characterization, safety assessment, and quality control are necessary to reduce uncertainty and accelerate approval processes.

(v) Data-driven personalization and integration into clinical workflows. Although personalized nanomedicine is a major promise of the field, its implementation depends on reliable integration of molecular diagnostics, predictive modeling, and clinical decision systems. Bridging this gap will require not only technological advances but also infrastructure for data interoperability and validation.

Addressing these challenges will determine whether polymer nanoparticle systems remain a promising technology or become a foundational component of precision medicine. Progress will depend on tighter integration of materials science, clinical research, regulatory science, and data engineering.

Polymer nanoparticles are no longer solely a platform for innovation—they are approaching a point of selective clinical impact. Their future influence will not be defined by their theoretical versatility, but by the field’s ability to translate complexity into safe, scalable, and clinically meaningful solutions. In summary, polymer nanoparticles represent a transformative technology in modern medicine, offering versatile platforms for drug delivery, gene therapy, diagnostics, and regenerative applications. Their adaptability, biocompatibility with many organisms, and capacity for functional customization position them as key drivers of next-generation therapeutics. As interdisciplinary collaboration continues to advance materials science, molecular biology, and clinical research, polymer nanoparticles are poised to redefine the landscape of medical treatment. The coming years will likely witness the emergence of smarter, safer, and more personalized nanoparticle-based interventions, shaping the future of healthcare in profound and unprecedented ways.

## Figures and Tables

**Table 1:** Literature search strategy and study selection criteria.

Category	Description
Study design	Narrative/structured literature review (or systematic review, if applicable)
Databases searched	PubMed, Scopus, Web of Science, ScienceDirect, and Google Scholar
Time frame	Publications from 2010 to 2026 (adjust depending on scope)
Language restriction	English-language publications only
Document types included	Peer-reviewed journal articles, review articles, book chapters, patents, and selected conference proceedings
Keywords used	“polymer-based nanoparticles”, “polymeric nanoparticles”, “drug delivery systems”, “controlled release nanoparticles”, “stimuli-responsive nanoparticles”, “targeted drug delivery”, “nanotheranostics”, “nanomedicine”, “polymeric micelles”, “polymer nanoparticles”, “lipid-polymer hybrid nanoparticles”, “personalized nanomedicine”, “AI drug delivery design”
Search strategy (example Boolean logic)	(“polymer-based nanoparticles” OR “polymeric nanoparticles” OR “polymeric nanoparticles”) AND (“drug delivery” OR “controlled release” OR “targeted therapy”) AND (theranostics OR “stimuli-responsive” OR “personalized medicine”)
Inclusion criteria	(1) Studies focused on polymer-based or polymeric hybrid nanoparticles; (2) articles addressing drug delivery, diagnostics, or theranostics applications; (3) studies reporting experimental, preclinical, or clinical data; (4) articles discussing design, functionalization, or biomedical applications; (5) publications within defined time frame.
Exclusion criteria	(1) Studies unrelated to biomedical or drug delivery applications; (2) non-polymeric inorganic nanoparticle systems unless directly compared; (3) editorials, opinions, and non-peer-reviewed articles (unless used for context); (4) duplicate publications; (5) articles without full-text availability.

**Table 2:** Summary of polymer nanoparticles used in imaging techniques.

Imaging Modality	Type of Polymer Nanoparticle System	Imaging Agent Incorporated	Main Clinical/Preclinical Applications	Role in Early Disease Detection	Main Advantages	Ref.
Magnetic Resonance Imaging (MRI)	poly(lactic-*co*-glycolic acid) (PLGA)-based nanoparticles	Gadolinium (Gd^3+^) chelates	Tumor imaging, brain disorders, vascular imaging	Detects small tumors via EPR effect; targeted imaging of early lesions	Reduced Gd toxicity, prolonged circulation, high payload capacity	[38]
Magnetic Resonance Imaging (MRI)	Polymer-coated iron oxide nanoparticles	Superparamagnetic iron oxide nanoparticles (SPIONs)	Liver imaging, inflammation, atherosclerosis	Detects macrophage accumulation in early plaques	Improved stability, enhanced targeting capability	[39]
Magnetic Resonance Imaging (MRI)	PEGylated polymeric micelles	Manganese (Mn^2+^)-based contrast agents	Early detection of neurodegenerative changes	Evaluates therapeutic distribution in central nervous system (CNS)	Lower toxicity than Gd systems, improved CNS penetration	[38]
Fluorescence Imaging	Dye-encapsulated polymer nanospheres	Rhodamine, fluorescein isothiocyanate, near-infrared (NIR) dyes	Tumor detection, infection imaging	Detects early-stage tumors with high sensitivity	Protection from photobleaching, strong signal amplification	[40]
Fluorescence Imaging	Conjugated polymer nanoparticles (polymer dots)	Intrinsically fluorescent polymers	Cellular imaging, molecular diagnostics	Visualizes early molecular changes	High brightness, superior photostability	[41]
Fluorescence Imaging (NIR)	PEGylated NIR polymer nanoparticles	Near-infrared fluorophores	Image-guided surgery, cancer diagnostics	Detects deeply located tumors	Low background noise, deeper penetration	[42]
Multimodal Imaging (MRI + Fluorescence)	Polymer nanoparticles	Gd^3+^ or SPIONs + fluorescent dyes	Oncology, neurology, theranostics	Early tumor detection with anatomical + molecular validation	Cross-validation, high sensitivity + high resolution	[43]
Stimulus-Responsive Imaging Nanoparticles	pH- or enzyme-responsive polymer nanoparticles	Activatable fluorophores or MRI agents	Tumor microenvironment imaging	Detects early biochemical abnormalities	High specificity, reduced background signal	[44]

**Table 3:** Role of artificial intelligence in theranostics.

Field	AI Techniques Used	Application in Theranostics	Clinical Impact	Ref.
Molecular Imaging Analysis	Convolutional neural networks (CNNs), deep learning	Automated lesion detection in PET/CT, PET/MRI, SPECT	Improved diagnostic accuracy and reduced inter-observer variability	[63]
Radiomics and Feature Extraction	Machine learning (Random Forest, SVM), deep learning	Extraction of quantitative imaging biomarkers	Enhanced tumor characterization and risk stratification	[64]
Patient Selection for Targeted Therapy	Predictive modeling, multivariate regression	Identification of candidates for radionuclide therapy	Optimized personalized treatment decisions	[65]
Treatment Response Prediction	Deep neural networks, survival models	Prediction of progression-free and overall survival	Early therapy modification and adaptive treatment planning	[66]
Dosimetry Optimization	Voxel-based AI segmentation, reinforcement learning	Organ-at-risk segmentation and absorbed dose estimation	Improved therapeutic index and reduced toxicity	[67]
Drug/Nanoparticle Design	Materials informatics, neural networks	Prediction of polymer–drug compatibility and release kinetics	Accelerated theranostic agent development	[68]
Workflow Automation	NLP, image recognition	Automated report generation and data integration	Increased efficiency and reduced clinician workload	[69]
Multimodal Data Integration	Multi-omics AI models, federated learning	Integration of imaging, genomic, and clinical data	Comprehensive precision medicine approach	[70]

**Table 4:** Comparison of stimulus-responsive drug delivery systems based on trigger source, advantages, limitations, and translational relevance [95,96,97,102,103,104,106,107].

Stimulus	Source	Advantages	Limitations	Clinical Relevance
pH	Tumor/endosomal acidity	Simple, well established	Limited specificity alone	High
Redox	Intracellular glutathione	Good intracellular selectivity	Variable tissue distribution	Medium–high
Enzyme	Disease-related enzymes	High specificity	Heterogeneous expression	Medium
Temperature	External heating	Controllable activation	Risk of overheating	Medium
Light	External irradiation	High precision	Limited penetration	Low–medium
Magnetic field	External field	Deep-tissue targeting	Equipment required	Medium

**Table 5:** Enzyme-responsive polymeric nanoparticle systems for targeted drug delivery.

Enzyme Trigger	Disease Context	Biological Role	Representative Polymer System	Activation Mechanism	References
MMP-2/MMP-9	Cancer	Extracellular matrix (ECM) degradation, tumor invasion	PEG–PLGA nanoparticles; peptide-cleavable liposomes	Peptide cleavage/PEG shedding/nanoparticle disassembly	[155,156,157]
Cathepsin B	Cancer, inflammation	Lysosomal protease activity	Polymer micelles with peptide cores	Endosomal cleavage/intracellular drug release	[144,158]
Hyaluronidase	Tumors, inflammation	Hyaluronic acid (HA) degradation	HA-based nanoparticles (CD44 targeting)	Glycosidic bond cleavage/matrix breakdown	[159,160]
Elastase	Chronic obstructive pulmonary disease inflammation	Neutrophil protease	Peptide-crosslinked nanogels	Crosslink cleavage/structural collapse	[161]
β-Glucuronidase	Tumor hypoxia	Lysosomal enzyme release	Glucuronide-linked prodrugs	Hydrolysis/drug activation	[162,163]
Dextranase	Infection	Bacterial polysaccharide degradation	Dextran-coated nanoparticles	Shell degradation/site-specific release	[164]
Lysozyme	Infection, inflammation	Cell wall polysaccharide hydrolysis	Chitosan nanoparticles	Glycosidic cleavage/polymer degradation	[165]
Lipases	Infection	Lipid hydrolysis	Lipid–polymer hybrids	Ester bond cleavage/destabilization	[166]
Esterases	Broad pathological conditions	Ester hydrolysis	PLGA-based systems	Accelerated ester cleavage/polymer erosion	[167]
Multi-enzyme systems	Solid tumors	Synergistic proteolysis	Multi-responsive micelles	Sequential enzymatic cleavage/stepwise disassembly	[168,169]

**Table 6:** Recent nanosponge applications [213,215,216,217].

Application Area	Description	Main Advantages
Drug detoxification/overdose management	Nanosponges bind excess drugs or toxins in bloodstream to reduce toxicity	Direct toxin removal, rapid action, reduced systemic side effects
Targeted cancer therapy	Delivery of chemotherapeutics (e.g., paclitaxel and doxorubicin) with controlled release	Improved bioavailability, reduced off-target toxicity, sustained release
Improved drug solubility and bioavailability	Encapsulation of poorly soluble drugs (hydrophobic compounds)	Enhanced dissolution, increased therapeutic efficacy
Controlled and sustained drug release	Programmable release for chronic therapies	Reduced dosing frequency, stable plasma drug levels
Antiviral applications (e.g., SARS-CoV-2)	Nanosponges act as decoys or delivery systems for antivirals	Viral neutralization, novel vaccine/drug platforms
Blood purification/toxin removal	Removal of uremic toxins and metabolic waste	Reduced organ burden, dialysis support potential
Neurological drug delivery	Crossing blood–brain barrier for CNS drugs	Targeted brain delivery, improved neurotherapy
Wound healing and tissue regeneration	Delivery of growth factors and antimicrobials	Controlled release, infection prevention, faster healing
Dermatological/topical delivery	Skin drug delivery and cosmetic applications	Reduced irritation, prolonged action, targeted penetration

## Data Availability

No new data were created or analyzed in this study.

## References

[1] Montaño-Grijalva E.A., Rodríguez-Félix F., Tapia-Hernández J.A., Márquez-Ríos E., Del-Toro-Sánchez C.L., Rodríguez-Félix D.E., Nalda-Molina R., Carvajal-Millan E., Barreras-Urbina C.G., López-Peña I.Y. (2026). Nano-Technology for Metformin Release Systems: Nanostructures, Biopolymer Carriers, and Techniques—A Review. Sci. Pharm..

[2] Francisco R.F., Azaret M.G.E., Enrique M.R., Silva J.M., Pérez J.A. (2026). Zein/Gelatin Coaxial Nanofibers for Metformin Encapsulation: Assessment on Composition, Structure, and Physicochemical Properties. Polym. Bull..

[3] Wen F., Wang L., Li X., Zhao J., Xu T., Zhu J., Ma L., Wang X. (2025). Precision Nanomedicine for Cancer: Innovations, Strategies, and Translational Challenges. OncoTargets Ther..

[4] Petros R.A., DeSimone J.M. (2010). Strategies in the Design of Nanoparticles for Therapeutic Applications. Nat. Rev. Drug Discov..

[5] El-Tanani M., Satyam S.M., Rabbani S.A., El-Tanani Y., Aljabali A.A.A., Al Faouri I., Rehman A. (2025). Revolutionizing Drug Delivery: The Impact of Advanced Materials Science and Technology on Precision Medicine. Pharmaceutics.

[6] Danhier F., Ansorena E., Silva J.M., Coco R., Le Breton A., Préat V. (2012). PLGA-Based Nanoparticles: An Overview of Biomedical Applications. J. Control. Release.

[7] Geszke-Moritz M., Moritz M. (2024). Biodegradable Polymeric Nanoparticle-Based Drug Delivery Systems: Overview and Challenges. Polymers.

[8] Yang M., Li J., Gu P., Fan X. (2021). The Application of Nanoparticles in Cancer Immunotherapy: Targeting Tumor Microenvironment. Bioact. Mater..

[9] Lionadi I., Payam A.F. (2026). Nanotechnology in Cancer Therapy: How Nanoparticles Are Shaping the Future of Personalized Treatment. ACS Nano Med..

[10] Maeda H., Nakamura H., Fang J. (2013). The EPR Effect for Macromolecular Drug Delivery to Solid Tumors: Improvement of Tumor Uptake, Lowering of Systemic Toxicity, and Distinct Tumor Imaging In Vivo. Adv. Drug Deliv. Rev..

[11] Fang J., Islam W., Maeda H. (2020). Exploiting the Dynamics of the EPR Effect and Strategies to Improve the Therapeutic Effects of Nanomedicines by Using EPR Effect Enhancers. Adv. Drug Deliv. Rev..

[12] Danhier F., Feron O., Préat V. (2010). Targeting of Tumor Endothelium by Nanoparticles. J. Control. Release.

[13] Bertrand N., Wu J., Xu X., Kamaly N., Farokhzad O.C. (2014). Cancer Nanotechnology: The Impact of Passive and Active Targeting. Mol. Pharm..

[14] Kelarakis A. (2024). In Situ Generation of Nanoparticles on and within Polymeric Materials. Polymers.

[15] La Mesa C. (2024). Hybrid Colloids Made with Polymers. Appl. Sci..

[16] Prasad Rao J., Geckeler K.E. (2011). Polymer Nanoparticles: Preparation Techniques and Size-Control Parameters. Prog. Polym. Sci..

[17] Prasad A.S., Wang Y., Li X., Iyer A., Chen W., Brinson L.C., Schadler L.S. (2020). Investigating the Effect of Surface Modification on the Dispersion Process of Polymer Nanocomposites. Nanocomposites.

[18] Mahlangu O.T., Motsa M.M., Richards H., Mamba B.B., George M.J., Nthunya L.N. (2024). The Impact of Nanoparticle Leach on Sustainable Performance of the Membranes—A Critical Review. Environ. Nanotechnol. Monit. Manag..

[19] Ehtezazi T., Sarker S.D. (2025). Phytochemical Nanoparticles for the Treatment of Neurological Disorders. Phytochem. Anal..

[20] Rai R., Alwani S., Badea I. (2019). Polymeric Nanoparticles in Gene Therapy: New Avenues of Design and Optimization for Delivery Applications. Polymers.

[21] Rauf M.A., Rao A., Sivasoorian S.S., Iyer A.K. (2025). Nanotechnology-Based Delivery of CRISPR/Cas9 for Cancer Treatment: A Comprehensive Review. Cells.

[22] Srivastava A., Ahmad A., Siddiqui S., Islam A. (2026). Innovations in Targeted Drug Delivery: From Nanotechnology to Clinical Applications. Next Nanotechnol..

[23] Shanahan K., Coen D., Nafo W. (2025). Polymer-Based Nanoparticles for Cancer Theranostics: Advances, Challenges, and Future Perspectives. Explor. BioMat-X.

[24] Ventola C.L. (2017). The Nanomedicine Revolution: Part 1: Emerging Concepts. Pharm. Ther..

[25] Parvin N., Aslam M., Alam M.N., Mandal T.K. (2025). Nanotechnology Driven Innovations in Modern Pharmaceutics: Therapeutics, Imaging, and Regeneration. Nanomaterials.

[26] Peer D., Karp J.M., Hong S., Farokhzad O.C., Margalit R., Langer R. (2007). Nanocarriers as an Emerging Platform for Cancer Therapy. Nat. Nanotechnol..

[27] Elsabahy M., Heo G.S., Lim S.M., Sun G., Wooley K.L. (2015). Polymeric Nanostructures for Imaging and Therapy. Chem. Rev..

[28] Liu M., Anderson R.C., Lan X., Conti P.S., Chen K. (2020). Recent Advances in the Development of Nanoparticles for Multimodality Imaging and Therapy of Cancer. Med. Res. Rev..

[29] Ryvolova M., Chomoucka J., Drbohlavova J., Kopel P., Babula P., Hynek D., Adam V., Eckschlager T., Hubalek J., Stiborova M. (2012). Modern Micro and Nanoparticle-Based Imaging Techniques. Sensors.

[30] Braeken Y., Cheruku S., Ethirajan A., Maes W. (2017). Conjugated Polymer Nanoparticles for Bioimaging. Materials.

[31] Wu C., Chiu D.T. (2013). Highly Fluorescent Semiconducting Polymer Dots for Biology and Medicine. Angew. Chem. Int. Ed..

[32] Choi H.S., Frangioni J.V. (2010). Nanoparticles for Biomedical Imaging: Fundamentals of Clinical Translation. Mol. Imaging.

[33] Arms L., Smith D.W., Flynn J., Palmer W., Martin A., Woldu A., Hua S. (2018). Advantages and Limitations of Current Techniques for Analyzing the Biodistribution of Nanoparticles. Front. Pharmacol..

[34] Mishra V., Kumari N., Vyas M., Aljabali A.A.A., Chattaraj A., Mishra Y. (2025). Advances in Multimodal Imaging Techniques in Nanomedicine: Enhancing Drug Delivery Precision. RSC Adv..

[35] Ilosvai Á.M., Forgách L., Kovács N., Heydari F., Szigeti K., Máthé D., Kristály F., Daróczi L., Kaleta Z. (2023). Development of Polymer Encapsulated, Amine Functionalized Zinc Ferrite Nanoparticles as MRI Contrast Agents. Int. J. Mol. Sci..

[36] Xu H., Yu P., Bandari R.P., Smith C.J., Aro M.R., Singh A., Ma L. (2024). Bimodal MRI/Fluorescence Nanoparticle Imaging Contrast Agent Targeting Prostate Cancer. Nanomaterials.

[37] Moloney C., McKiernan E., Brougham D.F. (2024). Enhancing the MRI Contrast and Hyperthermic Properties of Magnetic Nanoflowers by Modulating Magnetisation Dynamics Using Poly(Butyl Cyanoacrylate) Shells. Colloids Surf. A.

[38] Łopuszyńska N., Węglarz W.P. (2023). Contrasting Properties of Polymeric Nanocarriers for MRI-Guided Drug Delivery. Nanomaterials.

[39] Gauger A.J., Hershberger K.K., Bronstein L.M. (2020). Theranostics Based on Magnetic Nanoparticles and Polymers: Intelligent Design for Efficient Diagnostics and Therapy. Front. Chem..

[40] Pu K., Chattopadhyay N., Rao J. (2016). Recent Advances of Semiconducting Polymer Nanoparticles in In Vivo Molecular Imaging. J. Control. Release.

[41] Qian C.G., Yu J.C., Chen Y., Hu Q.Y., Xiao X.Z., Sun W.J., Wang C., Feng P.J., Shen Q.D., Gu Z. (2017). Conjugated Polymer Nanomaterials for Theranostics. Acta Pharmacol. Sin..

[42] Omri N., Kumaravel V. (2026). Molecular Architectonics of Semiconducting Polymer Dots for Next-Generation NIR-II Fluorescence Bioimaging. Nano Today.

[43] Smolak B., Dynarowicz K., Bartusik-Aebisher D., Henrykowska G., Aebisher D., Guz W. (2026). Combining Fluorescence and Magnetic Resonance Imaging in Drug Discovery—A Review. Pharmaceuticals.

[44] Wang Q., Guo Z., Chen Z., Wang C., Yang Y., Shi Q., Gao Q., Li H., Zhang D., Liu Y. (2025). pH-Responsive Materials for Therapy and Precision Biomedical Imaging. Chem. Biomed. Imaging.

[45] Anani T., Rahmati S., Sultana N., David A.E. (2021). MRI-Traceable Theranostic Nanoparticles for Targeted Cancer Treatment. Theranostics.

[46] Hosseini S.M., Mohammadnejad J., Salamat S., Beiram Zadeh Z., Tanhaei M., Ramakrishna S. (2023). Theranostic Polymeric Nanoparticles as a New Approach in Cancer Therapy and Diagnosis: A Review. Mater. Today Chem..

[47] Hazarika D., Sarma S., Shankarishan P. (2024). Nanotechnology in Cancer Therapeutics, Diagnosis, and Management. BioTechnologia.

[48] Janib S.M., Moses A.S., MacKay J.A. (2010). Imaging and Drug Delivery Using Theranostic Nanoparticles. Adv. Drug Deliv. Rev..

[49] Robin M.P., Mabire A.B., Damborsky J.C., Thom E.S., Winzer-Serhan U.H., Raymond J.E., O’Reilly R.K. (2013). New Functional Handle for Use as a Self-Reporting Contrast and Delivery Agent in Nanomedicine. J. Am. Chem. Soc..

[50] Yeniterzi D., Calla I.B., Cevher S.C., Gulseren G., Soylemez S. (2025). Advancement of Self-Reporting Polymer Nanoparticles for Melanoma Therapy and Biosensing. ACS Appl. Polym. Mater..

[51] Huang F., Xie Z., Zhang Q., Zada S., Lin R., Deng Y., Liu Q., Chen H., Zhou H., Miao H. (2025). Recent Advances in Fluorescence Resonance Energy Transfer (FRET) Biosensors for Exosomes. Curr. Issues Mol. Biol..

[52] Baidoo I., Sarbadhikary P., George B.P. (2026). Emerging Light-Based Strategies in Cancer Theranostics: Photodynamic Therapy, Nanomedicine, and Precision Oncology. Cancer Treat. Res. Commun..

[53] Yan S., Na J., Liu X., Wu P. (2024). Different Targeting Ligands-Mediated Drug Delivery Systems for Tumor Therapy. Pharmaceutics.

[54] Fallatah M.M., Alradwan I., Alfayez N., Aodah A.H., Alkhrayef M., Majrashi M., Jamous Y.F. (2025). Nanoparticles for Cancer Immunotherapy: Innovations and Challenges. Pharmaceuticals.

[55] Diaz-Ruano A.B., Gomez-Jimenez E., Llamas-Jimenez G., Ramirez-Muñoz A., Espejo-Hijano P., Rubio-Navarro A., Picon-Ruiz M. (2025). Advances in the Use of Nanoparticles for Specific Cell-Target Delivery of Anti-Cancer Agents. Life Sci..

[56] Wilhelm S., Tavares A.J., Dai Q., Ohta S., Audet J., Dvorak H.F., Chan W.C.W. (2016). Analysis of Nanoparticle Delivery to Tumors. Nat. Rev. Mater..

[57] Shi J., Kantoff P.W., Wooster R., Farokhzad O.C. (2017). Cancer Nanomedicine: Progress and Challenges. Nat. Rev. Cancer.

[58] Gidwani B., Sahu V., Shah K., Chauhan N.S., Alomary M.N., Ansari M.A., Anand S. (2025). Nanotheranostics: Emerging Nanomachines as Pharmacotherapeutics. 3 Biotech.

[59] Currie G.M., Rohren E. (2025). The Role of Artificial Intelligence in Theranostics. J. Nucl. Med. Technol..

[60] Li M., Zhang L., Ullah Z., Zhang Y., Wei S., Huang L., Guo B. (2025). Artificial Intelligence-Assisted Theranostics for Brain Tumors: Advancements and Future Perspectives. View.

[61] Zhao L., Liu X., Deng X. (2025). AI-Engineered Multifunctional Nanoplatforms: Synergistically Bridging Precision Diagnosis and Intelligent Therapy in Next-Generation Oncology. J. Nanobiotechnol..

[62] Tiwari A., Widodo, Krisnawati D.I., Park J.H., Lee S.Y., Kim J.H. (2026). AI-Driven Nanomedicine for Cancer Theranostics. Mol. Cancer.

[63] Gillies R.J., Kinahan P.E., Hricak H. (2016). Radiomics: Images Are More than Pictures, They Are Data. Radiology.

[64] Lambin P., Leijenaar R.T.H., Deist T.M., Peerlings J., de Jong E.E.C., van Timmeren J., Sanduleanu S., Larue R.T.H.M., Even A.J.G., Jochems A. (2017). Radiomics: The Bridge between Medical Imaging and Personalized Medicine. Nat. Rev. Clin. Oncol..

[65] Belabaci Z., Sleiay M., Abdelshafi A., Otmani Z., Moubarak E.S., Amer F. (2025). Safety and Efficacy of Lutetium-177 PSMA Therapy for Metastatic Castration-Resistant Prostate Cancer: A Systematic Review and Meta-Analysis of Randomized Controlled Trials. Clin. Genitourin. Cancer.

[66] Hosny A., Parmar C., Quackenbush J., Schwartz L.H., Aerts H.J.W.L. (2018). Artificial Intelligence in Radiology. Nat. Rev. Cancer.

[67] Hirata K., Matsui Y., Yamada A., Fujioka T., Yanagawa M., Nakaura T., Ito R., Ueda D., Fujita S., Tatsugami F. (2024). Generative AI and Large Language Models in Nuclear Medicine: Current Status and Future Prospects. Ann. Nucl. Med..

[68] Han Y., Kim D.H., Pack S.P. (2025). Nanomaterials in Drug Delivery: Leveraging Artificial Intelligence and Big Data for Predictive Design. Int. J. Mol. Sci..

[69] Kuo S.-M., Tai S.-K., Lin H.-Y., Chen R.-C. (2025). Automated Clinical Trial Data Analysis and Report Generation by Integrating Retrieval-Augmented Generation (RAG) and Large Language Model (LLM) Technologies. AI.

[70] Khan S.N., Danishuddin N.A.N., Khan M.W.A., Guarnera L., Akhtar S.M.F. (2026). Multi-Modal AI in Precision Medicine: Integrating Genomics, Imaging, and EHR Data for Clinical Insights. Front. Artif. Intell..

[71] Panchpuri M., Painuli R., Kumar C. (2025). Artificial Intelligence in Smart Drug Delivery Systems: A Step toward Personalized Medicine. RSC Pharm..

[72] Vogenberg F.R., Barash C.I., Pursel M. (2010). Personalized Medicine: Part 1: Evolution and Development into Theranostics. Pharm. Ther..

[73] Collins F.S., Varmus H. (2015). A New Initiative on Precision Medicine. N. Engl. J. Med..

[74] Ashley E.A. (2015). The Precision Medicine Initiative: A New National Effort. JAMA.

[75] Jameson J.L., Longo D.L. (2015). Precision Medicine—Personalized, Problematic, and Promising. N. Engl. J. Med..

[76] Lal N., Rastogi V., Mishra R., Jahan S., Ali H., Bharadwaj S., Goel R., Hashmi R.R. (2026). Polymeric Nanoparticles: A Promising Pharmaceutical Approach for Advanced Drug Delivery Systems. Pharm. Nanotechnol..

[77] Jia Z., Li J., Gao L., Yang D., Kanaev A. (2023). Dynamic Light Scattering: A Powerful Tool for In Situ Nanoparticle Sizing. Colloids Interfaces.

[78] Noury H., Rahdar A., Romanholo Ferreira L.F., Jamalpoor Z. (2025). AI-Driven Innovations in Smart Multifunctional Nanocarriers for Drug and Gene Delivery: A Mini-Review. Crit. Rev. Oncol. Hematol..

[79] Rao L., Wang J., Chen Y., Li X., Zhang H., Liu Z., Huang X. (2024). Designing Nanotheranostics with Machine Learning. Nat. Nanotechnol..

[80] Sreenivasulu A., Reddy P., Kumar M., Singh A., Patel S., Verma R., Sharma N. (2022). A Comprehensive Revision on the Nanocarrier Drug Delivery Systems with Special Reference to Artificial Intelligence. Int. J. Health Sci..

[81] Carini C., Seyhan A.A. (2024). Tribulations and Future Opportunities for Artificial Intelligence in Precision Medicine. J. Transl. Med..

[82] Bhange M., Telange D. (2025). Convergence of Nanotechnology and Artificial Intelligence in the Fight against Liver Cancer: A Comprehensive Review. Discov. Oncol..

[83] Zhang J., Yang X., Chang Z., Zhu W., Ma Y., He H. (2025). Polymeric Nanocarriers for Therapeutic Gene Delivery. Asian J. Pharm. Sci..

[84] Khare P.S., Shaikh S.A., Havelikar U. (2026). Artificial Intelligence and Precision Medicine for Optimizing Patient Care: A Comprehensive Review. Intell. Hosp..

[85] Maeda H., Wu J., Sawa T., Matsumura Y., Hori K. (2000). Tumor Vascular Permeability and the EPR Effect. J. Control. Release.

[86] Sun R., Xiang J., Zhou Q., Piao Y., Tang J., Shao S., Zhou Z., Bae Y.H., Shen Y. (2022). The Tumor EPR Effect for Cancer Drug Delivery: Current Status, Limitations, and Alternatives. Adv. Drug Deliv. Rev..

[87] Sell M., Lopes A.R., Escudeiro M., Esteves B., Monteiro A.R., Trindade T., Cruz-Lopes L. (2023). Application of Nanoparticles in Cancer Treatment: A Concise Review. Nanomaterials.

[88] Thongpon P., Tang M., Cong Z. (2025). Peptide-Based Nanoparticle for Tumor Therapy. Biomedicines.

[89] Saraiva C., Praça C., Ferreira R., Santos T., Ferreira L., Bernardino L. (2016). Nanoparticle-Mediated Brain Drug Delivery. J. Control. Release.

[90] Kochman U., Sitka H., Kuźniar J., Stopa M., Wójtowicz P., Kaczmarczyk R., Chalimoniuk M., Węsierska M. (2026). Targeted Nanoparticles for Drug Delivery across the Blood–Brain Barrier in Early and Late Stages of Alzheimer’s Disease: A Review. Mol. Neurobiol..

[91] Mukai H., Ogawa K., Kato N., Kawakami S. (2022). Recent Advances in Lipid Nanoparticles for Delivery of Nucleic Acid, mRNA, and Gene Editing-Based Therapeutics. Drug Metab. Pharmacokinet..

[92] Hosseini-Kharat M., Bremmell K.E., Prestidge C.A. (2025). Why Do Lipid Nanoparticles Target the Liver? Understanding of Biodistribution and Liver-Specific Tropism. Mol. Ther. Methods Clin. Dev..

[93] Cheng Q., Wei T., Farbiak L., Johnson L.T., Dilliard S.A., Siegwart D.J. (2020). Selective Organ Targeting (SORT) Nanoparticles for Tissue-Specific mRNA Delivery. Nat. Nanotechnol..

[94] Liu Y., Si L., Jiang Y., Jiang S., Zhang X., Li S., Chen J., Hu J. (2025). Design of pH-Responsive Nanomaterials Based on the Tumor Microenvironment. Int. J. Nanomed..

[95] Pi Y., Ganabady K., Celiz A.D. (2025). Enzyme-Responsive Biomaterials for Biomedical Applications. Commun. Mater..

[96] Bae Y.H., Park K. (2011). Targeted Drug Delivery to Tumors: Myths, Reality and Possibility. J. Control. Release.

[97] Hou X., Zaks T., Langer R., Dong Y. (2021). Lipid Nanoparticles for mRNA Delivery. Nat. Rev. Mater..

[98] Kulkarni J.A., Cullis P.R., van der Meel R. (2018). Lipid Nanoparticles Enabling Gene Therapies: From Concepts to Clinical Utility. Nat. Nanotechnol..

[99] Akinc A., Maier M.A., Manoharan M., Fitzgerald K., Jayaraman M., Barros S., Ansell S., Du X., Hope M.J., Madden T.D. (2019). The Onpattro Story and the Clinical Translation of Nanomedicines Containing Nucleic Acid-Based Drugs. Nat. Nanotechnol..

[100] Sahay G., Querbes W., Alabi C., Eltoukhy A., Sarkar S., Zurenko C., Karagiannis E., Love K., Chen D., Zoncu R. (2013). Efficiency of siRNA Delivery by Lipid Nanoparticles Is Limited by Endocytic Recycling. Nat. Biotechnol..

[101] Harini A., Perumal I. (2025). Polymeric Nanomaterials as a Drug Delivery System for Anticancer and Antibacterial Infections: A Review. RSC Adv..

[102] Subramanian J., Padhy R., Arun J., Murthannagari V.R., Ganesh G. (2025). Stimuli-Responsive Drug Delivery Systems: Extensive Overview. Int. J. Appl. Pharm..

[103] Arruebo M., Fernández-Pacheco R., Ibarra M.R., Santamaría J. (2007). Magnetic Nanoparticles for Drug Delivery. Nano Today.

[104] Zhang Y., Coleman M., Brekken R.A. (2021). Perspectives on Hypoxia Signaling in Tumor Stroma. Cancers.

[105] Chen B., Dai W., He B., Zhang H., Wang X., Wang Y., Zhang Q. (2017). Current Multistage Drug Delivery Systems Based on the Tumor Microenvironment. Theranostics.

[106] Wang Y., Lin M., Fan T., Zhou M., Yin R., Wang X. (2025). Advances of Stimuli-Responsive Amphiphilic Copolymer Micelles in Tumor Therapy. Int. J. Nanomed..

[107] Hou J., Xue Z., Chen Y., Li J., Yue X., Zhang Y., Gao J., Hao Y., Shen J. (2025). Development of Stimuli-Responsive Polymeric Nanomedicines in Hypoxic Tumors and Their Therapeutic Promise in Oral Cancer. Polymers.

[108] Wang Y., Grainger D.W. (2021). Lyophilized Liposome-Based Parenteral Drug Development: Reviewing Complex Product Design Strategies and Current Regulatory Environments. Adv. Drug Deliv. Rev..

[109] Blanco E., Shen H., Ferrari M. (2015). Principles of Nanoparticle Design for Overcoming Biological Barriers to Drug Delivery. Nat. Biotechnol..

[110] Kim Y., Kwak J., Lim M., Lim S.Y., Chae S., Ha S.J., Won Y.W., Kim H.O., Lim K.S. (2025). Advances in PCL, PLA, and PLGA-Based Technologies for Anticancer Drug Delivery. Pharmaceutics.

[111] Ganda S., Jiang Y., Thomas D.S., Eliezar J., Stenzel M.H. (2016). Biodegradable Glycopolymeric Micelles Obtained by RAFT-Controlled Radical Ring-Opening Polymerization. Macromolecules.

[112] Ahmad Q., Mehdi S., Shaukat B., Siddique R., Asif M.T., Malik A., Khan S., Siddiqui R., Ahmed Khan N., Mehmood M.H. (2026). Multi-Stimuli Responsive Nanoparticles: Next-Generation Platforms for Smart Drug Delivery. OpenNano.

[113] Karimi M., Eslami M., Sahandi-Zangabad P., Mirab F., Farajisafiloo N., Shafaei Z., Ghosh D., Bozorgomid M., Dashkhaneh F., Hamblin M.R. (2016). pH-Sensitive Stimulus-Responsive Nanocarriers for Targeted Delivery of Therapeutic Agents. WIREs Nanomed. Nanobiotechnol..

[114] Deirram N., Zhang C., Kermaniyan S.S., Johnston A.P.R., Such G.K. (2019). pH-Responsive Polymer Nanoparticles for Drug Delivery. Macromol. Rapid Commun..

[115] Zhang X., Lin Y., Gillies R.J. (2010). Tumor pH and Its Measurement. J. Nucl. Med..

[116] Reyes-Ortega F., Aguilar M.R., San Román J. (2014). pH-Responsive Polymers: Properties, Synthesis and Applications. Smart Polymers and Their Applications.

[117] Tang H., Zhao W., Yu J., Li Y., Zhao C. (2019). Recent Development of pH-Responsive Polymers for Cancer Nanomedicine. Molecules.

[118] Nunziata G., Pollonio D., Lacroce E., Rossi F. (2025). Smart pH-Responsive Polymers in Biomedical Applications: Nanoparticles, Hydrogels, and Emerging Hybrid Platforms. Mater. Today Chem..

[119] Wang H., Huang Q., Chang H., Xiao J., Cheng Y. (2016). Stimuli-responsive dendrimers in drug delivery. Biomater. Sci..

[120] Banerjee S., Banerjee D., Ram V., Kulhari H., Pooja D., Saharan V.A., Singh A. (2026). Engineering Stimuli-Responsive Dendrimers for Drug Delivery: A 15-Year Review of Formulation Strategies and Routes of Administration. OpenNano.

[121] Chu S., Shi X., Tian Y., Gao F. (2022). pH-Responsive Polymer Nanomaterials for Tumor Therapy. Front. Oncol..

[122] Kesharwani P., Kumar V., Goh K.W., Gupta G., Alsayari A., Wahab S., Sahebkar A. (2025). PEGylated PLGA Nanoparticles: Unlocking Advanced Strategies for Cancer Therapy. Mol. Cancer.

[123] Munster P., Krop I.E., LoRusso P., Ma C., Siegel B.A., Shields A.F., Molnár I., Wickham T.J., Reynolds J., Campbell K. (2018). Safety and Pharmacokinetics of MM-302, a HER2-Targeted Antibody–Liposomal Doxorubicin Conjugate, in Patients with Advanced HER2-Positive Breast Cancer: A Phase 1 Dose-Escalation Study. Br. J. Cancer.

[124] Lyon P.C., Griffiths L.F., Lee J., Chung D., Carlisle R., Wu F., Middleton M.R., Gleeson F.V., Coussios C.C. (2017). Clinical trial protocol for TARDOX: A phase I study to investigate the feasibility of targeted release of lyso-thermosensitive liposomal doxorubicin (ThermoDox®) using focused ultrasound in patients with liver tumours. J. Ther. Ultrasound.

[125] ClinicalTrials.gov. Identifier: NCT06434064. NCT06434064.

[126] Wolff A.C., Wang M., Li H., Pins M.R., Pretorius F.J., Rowland K.M., Sparano J.A., Davidson N.E. (2010). Phase II trial of pegylated liposomal doxorubicin plus docetaxel with and without trastuzumab in metastatic breast cancer: Eastern Cooperative Oncology Group trial E3198. Breast Cancer Res. Treat..

[127] Mamot C., Wicki A., Hasler-Strub U., Riniker S., Li Q., Holer L., Bärtschi D., Zaman K., von Moos R., Dedes K.J. (2023). A Multicenter Phase II Trial of Anti-EGFR-Immunoliposomes Loaded with Doxorubicin in Patients with Advanced Triple Negative Breast Cancer. Sci. Rep..

[128] Sun X., Yu K., Zhou Y., Dong S., Hu W., Sun Y., Li Y., Xie J., Lee R.J., Sun F. (2021). Self-Assembled pH-Sensitive Polymeric Nanoparticles for the Inflammation-Targeted Delivery of Cu/Zn-Superoxide Dismutase. ACS Appl. Mater. Interfaces.

[129] Zhao J., Zhao M., Yu C., Zhang X., Liu J., Cheng X., Lee R.J., Sun F., Teng L., Li Y. (2017). Multifunctional Folate Receptor-Targeting and pH-Responsive Nanocarriers Loaded with Methotrexate for Treatment of Rheumatoid Arthritis. Int. J. Nanomed..

[130] Zheng J., Sun Y., Shen Y., Zhou Z. (2025). Surface Engineering of Nanoparticles for Precision Medicine. Precis. Med. Eng..

[131] Zhang X., Han L., Liu M., Wang K., Tao L., Wan Q., Wei Y. (2017). Recent Progress and Advances in Redox-Responsive Polymers as Controlled Delivery Nanoplatforms. Mater. Chem. Front..

[132] Fu S., Rempson C.M., Puche V., Zhao B., Zhang F. (2022). Construction of Disulfide-Containing Redox-Responsive Polymeric Nanomedicine. Methods.

[133] Guo X., Cheng Y., Zhao X., Luo Y., Chen J., Yuan W.E. (2018). Advances in redox-responsive drug delivery systems of tumor microenvironment. J. Nanobiotechnol..

[134] Xu L., Cao Y., Xu Y., Li R., Xu X. (2024). Redox-Responsive Polymeric Nanoparticle for Nucleic Acid Delivery and Cancer Therapy: Progress, Opportunities, and Challenges. Macromol. Biosci..

[135] Abed H.F., Abuwatfa W.H., Husseini G.A. (2022). Redox-Responsive Drug Delivery Systems: A Chemical Perspective. Nanomaterials.

[136] Tiwari S., Bhattacharya S. (2026). Understanding Advanced Poly(ethylene glycol)-Disulphide-Poly(lactic-co-glycolic acid) (PEG-SS-PLGA) Nanoparticles for Cutting-Edge Innovations and Applications in Smart Drug Delivery Systems and Targeted Cancer Therapy. Biomed. Mater..

[137] Meng X., Shen Y., Zhao H., Liu Z., Wang J., Chen L., Zhang Y. (2024). Redox-Manipulating Nanocarriers for Anticancer Drug Delivery: A Systematic Review. J. Nanobiotechnol..

[138] Powell L.G., Alexander C., Stone V., Johnston H.J., Conte C. (2022). An In Vitro Investigation of the Hepatic Toxicity of PEGylated Polymeric Redox Responsive Nanoparticles. RSC Adv..

[139] Liu S., Yang J., Guo R., Deng L., Dong A., Zhang J. (2020). Facile Fabrication of Redox-Responsive Covalent Organic Framework Nanocarriers for Efficiently Loading and Delivering Doxorubicin. Macromol. Rapid Commun..

[140] Quinn J.F., Whittaker M.R., Davis T.P. (2017). Glutathione responsive polymers and their application in drug delivery systems. Polym. Chem..

[141] Ghosh D., Bag S., De P. (2026). Enzyme-Responsive Polymeric Materials with Anticancer and Antibacterial Activities. Biomacromolecules.

[142] Li M., Zhao G., Su W.K., Shuai Q. (2020). Enzyme-Responsive Nanoparticles for Anti-Tumor Drug Delivery. Front. Chem..

[143] Li J., Yu X., Huang D. (2026). Enzyme-Responsive Polymeric Drug Delivery Systems for the Treatment of Inflammatory Bowel Diseases: A Review. Polymers.

[144] Egorova V.S., Kolesova E.P., Lopus M., Yan N., Parodi A., Zamyatnin A.A. (2023). Smart Delivery Systems Responsive to Cathepsin B Activity for Cancer Treatment. Pharmaceutics.

[145] Xiao R., Zhou G., Wen Y., Ye J., Li X., Wang X. (2023). Recent Advances on Stimuli-Responsive Biopolymer-Based Nanocomposites for Drug Delivery. Compos. Part B Eng..

[146] Zong L., Xu H., Zhang H., Tu Z., Zhang X., Wang S., Li M., Feng Y., Wang B., Li L. (2024). A review of matrix metalloproteinase-2-sensitive nanoparticles as a novel drug delivery for tumor therapy. Int. J. Biol. Macromol..

[147] Hu Q., Katti P.S., Gu Z. (2014). Enzyme-Responsive Nanomaterials for Controlled Drug Delivery. Nanoscale.

[148] Kim J.H., Moon M.J., Kim D.Y., Heo S.H., Jeong Y.Y. (2018). Hyaluronic Acid-Based Nanomaterials for Cancer Therapy. Polymers.

[149] Eltaib L. (2025). Polymeric Nanoparticles in Targeted Drug Delivery: Unveiling the Impact of Polymer Characterization and Fabrication. Polymers.

[150] Mülhopt S., Diabaté S., Dilger M., Adelhelm C., Anderlohr C., Bergfeldt T., Gómez de la Torre J., Jiang Y., Valsami-Jones E., Langevin D. (2018). Characterization of Nanoparticle Batch-to-Batch Variability. Nanomaterials.

[151] Fernandes C., Jathar M., Sawant B.K.S., Warde T., Jindal A.B. (2023). Scale-Up of Nanoparticle Manufacturing Process. Pharmaceutical Process Engineering and Scale-Up Principles.

[152] Ventola C.L. (2017). Progress in Nanomedicine: Approved and Investigational Nanodrugs. Pharm. Ther..

[153] Rodriguez-Loya J., Lerma M., Gardea-Torresdey J.L. (2024). Dynamic Light Scattering and Its Application to Control Nanoparticle Aggregation in Colloidal Systems: A Review. Micromachines.

[154] Shrestha S., Wang B., Dutta P. (2020). Nanoparticle Processing: Understanding and Controlling Aggregation. Adv. Colloid Interface Sci..

[155] Rahban M., Ahmad F., Piatyszek M.A., Haertlé T., Saso L., Saboury A.A. (2023). Stabilization Challenges and Aggregation in Protein-Based Therapeutics in the Pharmaceutical Industry. RSC Adv..

[156] Wang Q., Cui H., Gan N., Ma X., Ren W., Wu A. (2022). Recent Advances in Matrix Metalloproteinases-Responsive Nanoprobes for Cancer Diagnosis and Therapy. Rev. Anal. Chem..

[157] Li X., Xu Z. (2025). Applications of Matrix Metalloproteinase-9-Related Nanomedicines in Tumors and Vascular Diseases. Pharmaceutics.

[158] Zhong Y., Meng F., Deng C., Zhong Z. (2014). Ligand-Directed Polymeric Micelles for Tumor-Targeted Drug Delivery. J. Control. Release.

[159] Wang Y., Kohane D.S. (2017). Enzyme-Responsive Nanoparticles for Drug Delivery. J. Control. Release.

[160] Fan Y., Zhang Q., Yang J., Zhao X., Li X., Wang L., Liu Y., Chen H. (2018). Hyaluronic Acid-Based Nanocarriers for Cancer Therapy. Carbohydr. Polym..

[161] Guo X., Shi C., Yang G., Wang J., Cai Z., Zhou S. (2014). Dual-Responsive Polymer Micelles for Target-Cell-Specific Anticancer Drug Delivery. Chem. Mater..

[162] Gao W., Chan J.M., Farokhzad O.C. (2010). pH- and Enzyme-Responsive Polymeric Nanoparticles for Cancer Therapy. J. Control. Release.

[163] Martin H., Ramírez Lázaro L., Gunnlaugsson T., Scanlan E.M. (2022). Glycosidase activated prodrugs for targeted cancer therapy. Chem. Soc. Rev..

[164] Amador-Gómez L.P., Luna Solano G., Urrea-García G.R., Gines-Palestino R.S., Cantú-Lozano D. (2023). Synthesis, Modification, and Characterization of Fe_3_O_4_@SiO_2_-PEI-Dextranase Nanoparticles for Enzymatic Degradation of Dextran in Fermented Mash. Processes.

[165] Gálvez-Iriqui A.C., Hoyos-Verdugo I.I., Argüelles-Monal W.M., Rosas-Durazo A.d.J., Burgos-Hernández A., López-Meneses A.K., Plascencia-Jatomea M. (2026). Chitosan–κ-Carrageenan–Lysozyme Nanoparticles Disrupt Appressorium Formation and Cellular Architecture in *Colletotrichum siamense* with Low Sensitivity to Chitosan. Polysaccharides.

[166] Gao Y., Kuang Y., Li J., Zhao D., Ge Z., Han X., Chen S., Wang Z. (2019). Enzyme-Sensitive Nanocarriers for Targeted Cancer Therapy. Adv. Drug Deliv. Rev..

[167] Siepmann J., Siepmann F. (2025). Release Mechanisms of PLGA-Based Drug Delivery Systems: A Review. Int. J. Pharm. X.

[168] Mura S., Nicolas J., Couvreur P. (2013). Stimuli-Responsive Nanocarriers for Drug Delivery. Nat. Mater..

[169] Zeng M., Yang Z., Yang L., Wang X., Wang Y., Chen T., Yin X., Wu F. (2026). Enzyme-Responsive Targeted Nanomedicines: A Novel Strategy for Cancer Therapy. J. Mater. Chem. B.

[170] Li J., Mooney D.J. (2016). Designing Hydrogels for Controlled Drug Delivery. Nat. Rev. Mater..

[171] Bernal-Chávez S.A., Del Prado-Audelo M.L., Caballero-Florán I.H., Giraldo-Gomez D.M., Figueroa-Gonzalez G., Reyes-Hernandez O.D., González-Del Carmen M., González-Torres M., Cortés H., Leyva-Gómez G. (2021). Insights into Terminal Sterilization Processes of Nanoparticles for Biomedical Applications. Molecules.

[172] Domańska I.M., Figat R., Zalewska A., Cieśla K., Kowalczyk S., Kędra K., Sobczak M. (2023). The Influence of Ionizing Radiation on Paclitaxel-Loaded Nanoparticles Based on PLGA. Appl. Sci..

[173] Rehman M., Tahir N., Sohail M.F., Qadri M.U., Duarte S.O.D., Brandão P., Esteves T., Javed I., Fonte P. (2024). Lipid-Based Nanoformulations for Drug Delivery: An Ongoing Perspective. Pharmaceutics.

[174] Javid-Naderi M.J., Mousavi Shaegh S.A. (2025). Advanced Microfluidic Techniques for the Preparation of Solid Lipid Nanoparticles: Innovations and Biomedical Applications. Int. J. Pharm. X.

[175] Hare J.I., Lammers T., Ashford M.B., Puri S., Storm G., Barry S.T. (2017). Challenges and Strategies in Anti-Cancer Nanomedicine Development. Adv. Drug Deliv. Rev..

[176] Desai N., Rana D., Patel M., Bajwa N., Prasad R., Vora L.K. (2025). Nanoparticle Therapeutics in Clinical Perspective: Classification, Marketed Products, and Regulatory Landscape. Small.

[177] Stucchi F., Li M., Castellano G., Cellesi F. (2026). Regulatory Framework for Polymer-Based Nanotherapeutics in Clinical Translation. Front. Bioeng. Biotechnol..

[178] Zhang M., Song W., Li X., Wang Y., Liu H., Chen Z., Zhao Y. (2022). Polymer-Based Nanofiber–Nanoparticle Hybrids and Their Medical Applications. Polymers.

[179] Nirwan V.P., Kowalczyk T., Bar J., Buzgo M., Filová E., Fahmi A. (2022). Advances in Electrospun Hybrid Nanofibers for Biomedical Applications. Nanomaterials.

[180] Vu T.N., Vitale A. (2026). Coupling of electrospinning and photo-induced processes for advanced nanofibrous polymeric materials: Current state-of-the-art and future perspectives. Soft Matter.

[181] Nirwan V.P., Kumar S., Singh R., Sharma A., Gupta N., Verma P., Jain A. (2025). Electrospun Smart Hybrid Nanofibers for Multifaceted Applications. Macromol. Rapid Commun..

[182] Camposeo A., Persano L., Pisignano D. (2018). Nanowire-Intensified MEF in Hybrid Electrospun Filaments. Small.

[183] Vargas-Molinero H.Y., García-García A., Sánchez-García M.D., Gómez-López J., Martínez-Pérez A., Rodríguez-Hernández J., García-García J.M. (2023). Hybrid Systems of Nanofibers and Polymeric Nanoparticles. Micromachines.

[184] Chou S.-F., Carson D., Woodrow K.A. (2015). Current strategies for sustaining drug release from electrospun nanofibers. J. Control. Release.

[185] Kutvonen A., Rossi M., Ala-Nissila T. (2012). Influence of Nanoparticle Size and Loading on Polymer Nanocomposites. J. Chem. Phys..

[186] Antunes M., Arencón D. (2026). Recent Developments in the Mechanical Behavior of Polymer-Based Composites. Polymers.

[187] Ahmad N., Bukhari S.N.A., Hussain M.A., Ejaz H., Munir M.U., Amjad M.W. (2024). Nanoparticles incorporated hydrogels for delivery of antimicrobial agents: Developments and trends. RSC Adv..

[188] Zhang X., Shi X., Gautrot J.E., Peijs T. (2021). Nanoengineered Electrospun Fibers and Their Biomedical Applications: A Review. Nanocomposites.

[189] Gupta N., Kamath S.M., Rao S.K., Jaison D., Patil S., Gupta N., Arunachalam K.D. (2021). Kaempferol Loaded Albumin Nanoparticles and Dexamethasone Encapsulation into Electrospun Polycaprolactone Fibrous Mat—Concurrent Release for Cartilage Regeneration. J. Drug Deliv. Sci. Technol..

[190] Chen K., Li Y., Li Y., Wang X., Zhang H., Liu Y., Zhao J. (2023). Stimuli-Responsive Electrospun Nanofibers for Drug Delivery, Cancer Therapy, Wound Dressing, and Tissue Engineering. J. Nanobiotechnol..

[191] Kabay G., Meydan A.E., Eom T., Shim B.S., Mutlu M., Kaleli-Can G. (2023). Stimuli-Responsive Nanoparticle–Nanofiber Hybrids for Drug Delivery and Photodynamic Therapy. Int. J. Pharm..

[192] Yousefzade O., Katsarava R., Puiggalí J. (2020). Biomimetic Hybrid Systems for Tissue Engineering. Biomimetics.

[193] El-Seedi H.R., Said N.S., Yosri N., Hawash H.B., El-Sherif D.M., Abouzid M., Abdel-Daim M.M., Yaseen M., Omar H., Shou Q. (2023). Gelatin Nanofibers: Recent Insights in Synthesis, Biomedical Applications and Limitations. Heliyon.

[194] Mohite P., Puri A., Munde S., Dave R., Khan S., Patil R., Singh A.K., Tipduangta P., Singh S., Chittasupho C. (2025). Potential of Chitosan/Gelatin-Based Nanofibers in Delivering Drugs for the Management of Varied Complications: A Review. Polymers.

[195] Abou-Dobara M.I., Baka Z.A.M., El-Salamony S.M., El-Zahed M.M. (2025). Enhanced antimicrobial efficacy of a vancomycin/zinc oxide/chitosan nanocomposite via *Bacillus licheniformis*-mediated biomodification. Discov. Nano.

[196] Pathak K., Sarma M., Sahariah M., Shankarishan P., Sahariah J.J., Deka S., Das A., Islam M.A., Pramanik P., Borthakur P.P. (2026). Nanoparticles in the fight against antimicrobial challenges: A comprehensive review. Next Nanotechnol..

[197] Wang Y., Zhang W., Karamergenova A., Lin L. (2025). Fabrication and Application of Polycaprolactone-Based Composite Scaffolds in Tissue Engineering: A Review. Mater. Today Commun..

[198] Liu S., Yu J.-M., Gan Y.-C., Qiu X.-Z., Gao Z.-C., Wang H., Chen S.-X., Xiong Y., Liu G.-H., Lin S.-E. (2023). Biomimetic natural biomaterials for tissue engineering and regenerative medicine: New biosynthesis methods, recent advances, and emerging applications. Mil. Med. Res..

[199] Yu D. (2022). Biomedical Applications of Nanofiber–Nanoparticle Hybrids. Encyclopedia.

[200] Habibzadeh F., Sadraei S.M., Mansoori R., Chauhan N.P.S., Sargazi G. (2022). Nanomaterials Supported by Polymers for Tissue Engineering Applications: A Review. Heliyon.

[201] Zhou Q., Lyu S., Bertrand A.A., Hu A.C., Chan C.H., Ren X., Dewey M.J., Tiffany A.S., Harley B.A.C., Lee J.C. (2021). Stiffness of Nanoparticulate Mineralized Collagen Scaffolds Triggers Osteogenesis via Mechanotransduction and Canonical Wnt Signaling. Macromol. Biosci..

[202] Boateng J.S., Matthews K.H., Stevens H.N.E., Eccleston G.M. (2015). Wound healing dressings and drug delivery systems. J. Pharm. Sci..

[203] Yusuf Aliyu A., Adeleke O.A. (2023). Nanofibrous Scaffolds for Diabetic Wound Healing. Pharmaceutics.

[204] Guo B., Ma P.X. (2018). Conducting Polymers for Tissue Engineering. Biomacromolecules.

[205] Gu X., Ding F., Williams D.F. (2014). Neural Tissue Engineering Options for Peripheral Nerve Regeneration. Biomaterials.

[206] Pashneh-Tala S., MacNeil S., Claeyssens F. (2016). Small-Diameter Vascular Grafts: Current Status and Future Directions. Acta Biomater..

[207] Tabish T.A., Crabtree M.J., Townley H.E., Winyard P.G., Lygate C.A. (2024). Nitric oxide releasing nanomaterials for cardiovascular applications. JACC Basic Transl. Sci..

[208] Garg A., Lai W.-C., Chopra H., Agrawal R., Singh T., Chaudhary R., Dubey B.N. (2024). Nanosponge: A Promising and Intriguing Strategy in Medical and Pharmaceutical Science. Heliyon.

[209] Rao M.R.P., Chaudhari J., Trotta F., Caldera F., Rocco F., Savi S., Cavalli R. (2018). Investigation of Cyclodextrin-Based Nanosponges for Solubility and Bioavailability Enhancement of Rilpivirine. AAPS PharmSciTech.

[210] Omar S.M., Ibrahim F., Ismail A., Youssef N.A.H.A., Abdallah M.H., El-Helw A.R. (2020). Formulation and Evaluation of Cyclodextrin-Based Nanosponges of Griseofulvin as Pediatric Oral Liquid Dosage Form for Enhancing Bioavailability and Masking Bitter Taste. Saudi Pharm. J..

[211] Swaminathan S., Vavia P., Trotta F., Cavalli R., Rocco F., Rossi S. (2007). Formulation of β-Cyclodextrin-Based Nanosponges of Itraconazole. J. Incl. Phenom. Macrocycl. Chem..

[212] Koppula S., Maddi S. (2024). Nanosponges in Therapeutics: Current Advancements and Future Directions in Targeted Drug Delivery. J. Drug Deliv. Sci. Technol..

[213] Shah A.A., Kehinde E.O., Patel J. (2021). An Emerging Era for Targeted Drug Delivery: Nanosponges. J. Pharm. Res. Int..

[214] Sakhuja N., Khushi, Jain C., Chauhan S.B., Singh I. (2025). Nanosponges in Detoxification: Strategy for Toxin Removal and Drug Overdose Management. Recent Adv. Drug Deliv. Formul..

[215] Hu C.M., Fang R.H., Copp J., Luk B.T., Zhang L. (2013). A Biomimetic Nanosponge that Absorbs Pore-Forming Toxins. Nat. Nanotechnol..

[216] Girigoswami A., Girigoswami K. (2022). Versatile Applications of Nanosponges in Biomedical Field: A Glimpse on SARS-CoV-2 Management. Bionanoscience.

[217] Sharma A., Krishan O., Sharma N., Surya A., Gautam V. (2024). Nanoguard: Revolutionizing Skin Care with Topical Nanosponges—A Novel Approach to Combat Skin Infections. J. Appl. Pharm. Sci. Res..

[218] Meshram S.I., Hatwar P.R., Bakal R.L., Rotake S.B. (2024). An outlook towards nano-sponges: A unique drug delivery system and its application in drug delivery. GSC Biol. Pharm. Sci..

[219] Caldera F., Tannous M., Cavalli R., Zanetti M., Trotta F. (2017). Evolution of Cyclodextrin Nanosponges. Int. J. Pharm..

[220] Pyrak B., Rogacka-Pyrak K., Gubica T., Szeleszczuk Ł. (2024). Exploring Cyclodextrin-Based Nanosponges as Drug Delivery Systems: Understanding the Physicochemical Factors Influencing Drug Loading and Release Kinetics. Int. J. Mol. Sci..

[221] U.S. Food and Drug Administration (FDA) GRAS Notice Inventory. https://www.fda.gov.

[222] Idrees H., Zaidi S.Z.J., Sabir A., Khan R.U., Zhang X., Hassan S.U. (2020). A Review of Biodegradable Natural Polymer-Based Nanoparticles for Drug Delivery Applications. Nanomaterials.

[223] Kean T., Thanou M. (2010). Biodegradation, Biodistribution and Toxicity of Chitosan. Adv. Drug Deliv. Rev..

[224] Makadia H.K., Siegel S.J. (2011). Poly Lactic-co-Glycolic Acid (PLGA) as Biodegradable Controlled Drug Delivery Carrier. Polymers.

[225] Woodruff M.A., Hutmacher D.W. (2010). The Return of a Forgotten Polymer—Polycaprolactone in the 21st Century. Prog. Polym. Sci..

[226] Dash T.K., Konkimalla V.B. (2012). Poly-ε-caprolactone Based Formulations for Drug Delivery and Tissue Engineering: A Review. J. Control. Release.

[227] Abdulsalam L., Abubakar S., Permatasari I., Lawal A.A., Uddin S., Ullah S., Ahmad I. (2025). Advanced Biocompatible and Biodegradable Polymers: A Review of Functionalization, Smart Systems, and Sustainable Applications. Polymers.

[228] Vert M., Doi Y., Hellwich K.H., Hess M., Hodge P., Kubisa P., Rinaudo M., Schué F. (2012). Terminology for biorelated polymers and applications (IUPAC Recommendations 2012). Pure Appl. Chem..

[229] European Bioplastics (2021). Bioplastics and Life Cycle Assessment in Sustainable Materials Systems. Sustainability.

[230] Sharma P., Kaur H., Kaur G., Sharma S. (2022). Biodegradable Polymers for Sustainable Biomedical Applications. Polymers.

[231] Pelaz B., Alexiou C., Alvarez-Puebla R.A., Alves F., Andrews A.M., Ashraf S., Balogh L.P., Ballerini L., Bestetti A., Brendel C. (2017). Diverse Applications of Nanoparticles in Nanomedicine: Biodegradable Systems and Safety Considerations. ACS Nano.

[232] Kyriakides T.R., Raj A., Tseng T.H., Xiao H., Nguyen R., Mohammed F.S., Halder S., Xu M., Wu M.J., Bao S. (2021). Biocompatibility of nanomaterials and their immunological properties. Biomed. Mater..

[233] Niaounakis M. (2013). Biopolymers: Reuse, Recycling, and Disposal.

[234] United Nations (2015). Transforming Our World: The 2030 Agenda for Sustainable Development. https://sustainabledevelopment.un.org/post2015/transformingourworld/publication.

